# Anxiety in Young Children with Williams Syndrome: A Longitudinal Study

**DOI:** 10.3390/children12081098

**Published:** 2025-08-21

**Authors:** Jessica L. Reeve, Melanie A. Porter

**Affiliations:** School of Psychological Sciences, Macquarie University, Sydney 2109, Australia

**Keywords:** Williams syndrome, preschool, anxiety

## Abstract

**Highlights:**

**What are the main findings?**

• Anxiety symptomology is highly prevalent in very young children with Williams syndrome, and seems to increase with age/over time (with many children developing co-occurring anxiety disorder symptoms).
• Our longitudinal findings also provide evidence for the contribution of environmental factors on the nature, developmental course, and maintenance of anxiety, notably, chronological age, sex, and IQ.

**What is the implication of the main finding?**

• The importance of screening the Williams syndrome population at a very early age and allows clinicians to identify those most at risk of developing an anxiety disorder.
• The existing anxiety classification systems should be reviewed to reflect specific symptomology in young children with intellectual disabilities or delayed verbal skills, and implications for early intervention programmes aimed at reducing anxiety risk in Williams syndrome individuals.

**Abstract:**

**Background/Objectives**: Anxiety is a hallmark feature of Williams syndrome (WS), with very high prevalence rates of generalised anxiety disorder (GAD) and specific phobias in both school-aged children and adults, yet a relatively lower prevalence of social phobia. There is very limited research on anxiety in very young children with WS, and no study to date has examined the early prevalence and development of different anxiety disorders in WS. The present research provides a comprehensive assessment of the prevalence and longitudinal profile of anxiety symptomology in very young children with WS. Potential environmental and demographic correlates of anxiety symptomology were also explored. **Methods**: Participants included 19 young children with WS, aged between 2 and 5 years (at initial testing), who completed a comprehensive developmental assessment. Parents/guardians also completed the Spence Children’s Anxiety Scale (SCAS; Spence, 1997 & Spence et al., 2001), a standardised, psychometrically robust anxiety questionnaire (commonly utilised in research and clinical settings) that measures anxiety symptomology for various anxiety disorders present in the Diagnostic and Statistical Manual of Mental Disorders, Fifth Edition (DSM-5; American Psychiatric Association, 2013). **Results**: The present research found anxiety symptomology to be highly prevalent in very young children with WS, particularly GAD and specific phobia. Moreover, the prevalence of anxiety symptomology increased with age and over time, with many children developing comorbid anxiety disorder symptoms approximately 3.5 years later, at Time 2. Chronological age, sex, and developmental/intellectual capabilities were also found to impact on the developmental trajectory of anxiety in young children with WS. **Conclusions**: The longitudinal findings provide evidence for the contribution of environmental factors on the nature, developmental course, and maintenance of anxiety. Considerable individual variability was apparent, confirming the importance of individual assessments and developing individualised treatment programmes for those with WS.

## 1. Anxiety in Young Children with Williams Syndrome: A Longitudinal Study

Williams syndrome (WS) is a genetic condition that affects cognitive, emotional and behavioural functioning. Anxiety is extremely common in WS; however, the early trajectory of anxiety in WS is not yet well understood. To date, although there appears to be a strong genetic link between anxiety and the genes implicated in WS, no longitudinal research has focused on environmental contributions to the development and maintenance of anxiety in WS. Approximately 45 to 65 percent of WS individuals will experience an anxiety disorder across their lifespan [1], with the prevalence reportedly increasing with age, at least from late childhood to adulthood [2,3,4]. Anxiety in WS and in the general population often results in a significantly impaired quality of life, family and social dysfunction, reduced recreational opportunity and engagement, increased parental and family stress, financial strain, and often the need for psychological and/or pharmacological intervention [5,6,7,8,9]. Within the general population, anxiety in childhood is also associated with reduced academic performance, victimisation, and bullying [10,11], low self-esteem [12], and other mental health difficulties such as depression [13,14]. These effects have been shown to continue into adulthood [15,16]. Early intervention and preventative measures are key in reducing anxiety. The present study offers a unique opportunity to better extend our knowledge of anxiety in the very young WS population given their striking predisposition for anxiety and other mental health disorders. Understanding the early trajectory of anxiety in WS will help to better understand the development and maintenance of anxiety, which can inform early intervention and, in the future, help to reduce the high prevalence of anxiety disorders in this population, who are biologically prone to anxiety.

There is a known biological risk factor for anxiety in WS. WS is caused by sporadic, submicroscopic, and multi-gene deletion of the long arm of chromosome 7 at 7q11.23, spanning approximately 26 to 28 genes [17]. Certain genes in the WS region have been linked to anxiety, not only in WS, but also in the general population [6,18,19]. The prevalence of anxiety is up to seven times higher in individuals with WS than the typically developing population (globally), and four times higher than the prevalence of anxiety in the intellectual disability (ID) population [20]. Further, the most commonly reported anxiety disorders in WS individuals are generalised anxiety disorder (GAD) and specific phobias, with a lower prevalence of social phobia [3,21]. The high rates of GAD and specific phobias in WS individuals suggests that specific anxiety disorders may be linked to the WS genetic aetiology [3]. Evidence for this biological vulnerability has also been demonstrated through numerous brain imaging studies, where functional alterations in the brain have been shown in WS, including a lack of brain response to social threat, together with increased non-social threat-related brain responses [22,23,24]. Studies have found that WS individuals show reduced amygdala activation in response to social threat [22,25] and reduced attention bias towards social threat experimentally [26]. Some research suggests structural alterations in white matter pathways may result in increased amygdala activation when WS individuals observe non-social threatening stimuli [24,27,28].

Studies have also shown a moderate genetic influence in the development of anxiety in young typically developing children [29]. More specifically, children with anxiety symptomatology are more likely to have a parent or family member with an anxiety disorder [30]. Twin studies also suggest moderate heritability of anxiety symptoms [31], with little difference in the genetic contributions across each specific anxiety disorder [3,32,33]. Although no specific genes for generalised anxiety or specific phobias have been identified thus far [34], the general transcription factor gene, GTF2I (hemizygously deleted from most WS individuals), has been implicated [35,36].

Although specific genes and other biological factors may predispose an individual to developing an anxiety disorder, environmental factors are also associated with the manifestation of an anxiety disorder, the types of anxiety experienced, and the maintenance of anxiety [37]. The most common types of specific phobias reported in the WS literature, for example, are loud noises, thunderstorms, going to the doctor/dentist, and blood tests/injections (e.g., [3,38,39]). It has been suggested that the WS phenotypic characteristics of hyperacusis (sensitivity to sound) and medical issues such as heart disease (resulting in frequent hospitalisations and medical interventions) may lead to a fear of loud noises and medical tests/visits [21], with one study finding an association between sensory hypersensitivity and increased levels of anxiety in adolescents and adults with WS [40]. No research to date, however, has explored the relationship between environmental contributions and the risk factors for the development of specific anxiety disorders in WS. Furthermore, there is little research focussing on anxiety in very young children with WS, even though much of the limited early data indicates that anxiety does manifest at this young age [41,42,43,44,45].

In the general population, there is growing evidence that points towards several early environmental risk factors for the onset, type, and maintenance of anxiety disorders. Specific learning experiences during early development have been suggested to likely contribute to the development of fears in young children [46,47], and stressful life events and trauma (e.g., risk of losing parent/caregiver, physical danger, witnessing a trauma, and considerable psychological challenges) have also been linked to anxiety [48]. Twin studies also link anxiety to select aspects of the environment [49], such as parent–child interactions, and parent anxiety and emotional distress [50,51]. Additionally, although considered a combination of environmental and genetic factors [52], a withdrawn, avoidant, and inhibited temperament is thought to be a central component of the development of an anxiety disorder, which may elicit and interact with other risk factors [53].

## 2. Anxiety in Williams Syndrome

Anxiety is a hallmark feature of WS and one of the most challenging difficulties experienced by these individuals and their families. A systematic review and meta-analysis on anxiety in WS [20] highlighted some methodological issues present in studies of anxiety in WS, including a reliance on symptom checklists and choice of comparative samples [20,26,38,54]. Royston and colleagues [20] indicated that the current ‘gold-standard’ assessment of anxiety should utilise psychometrically robust diagnostic measures that correspond to a clinical classification system (i.e., the Diagnostic and Statistical Manual of Mental Disorders, Fifth Edition [DSM-5] [55]; or the International Classification of Diseases [ICD] [56]).

However, anxiety symptom checklists are most commonly utilised in WS research, as they are efficient and practical, and some are of a higher quality diagnostically and psychometrically than others. This is particularly true in studies of young WS children, as diagnostic screening measures are an important tool when the functional impact of anxiety may or may not be recognised at this early age, depending on the environmental demands. Nevertheless, studies that have utilised both robust diagnostic interviews or standardised checklists based on DSM criteria (i.e., ‘gold-standard’ anxiety measures) have consistently found high prevalence rates for specific phobia and GAD in both children and adults with WS (e.g., [2,3,38,39]).

### 2.1. How Are Chronological Age, Sex, and IQ Related to Anxiety in WS?

Most cross-sectional and longitudinal research in WS to date suggests that anxiety increases with age. More specifically, these studies indicate that the prevalence rates of GAD are lower in primary school aged children and adolescents (aged 6 to 17 years) than in adults with WS [2,3,4]. Of these studies, however, it is unclear what factors were associated with these changes. Interestingly, no significant age-group differences were reported for the prevalence of specific phobia, suggesting that this aspect of anxiety is present in WS individuals from an early age and, perhaps, GAD is more likely with increased chronological age [2,3]. Furthermore, there is some evidence to suggest that anxiety disorders track a U-shaped trajectory across the lifespan in both WS (e.g., [2,38]) and in the general population (e.g., [57]). For example, it could be postulated that anxiety may increase in younger years, reduce in middle childhood, then increase again in late adolescence/early adulthood. This pattern may, at least partially, reflect times of greatest transition or change, for example, starting day-care/primary school and finishing high school. Of note, however, epidemiological studies in typically developing preschool children (aged approximately 3 to 5 years) have shown minimal changes in the prevalence of anxiety disorders across this young age [29].

In terms of sex differences in anxiety, findings are mixed in the WS literature. Although not statistically significant, Dodd and Porter [2] found that specific phobia and GAD appeared more common in females with WS, consistent with findings in the neurotypical population [29,57,58,59]. Of note, sex differences in symptoms of anxiety generally emerge from approximately 4- to 5 years of age in typically developing children [60]. Dykens [21] also found some evidence that specific fears (as measured by the Fear Survey Schedule for Children-Revised; [61]) may be more prevalent in WS females. In contrast, however, Leyfer et al. [3] found no relation between the presence of GAD or social phobia and sex. These discrepancies may be attributable to differences in sampling groups.

Similarly, there is variability in the literature pertaining to the association between anxiety and IQ in WS individuals. Dykens [21], Leyfer et al. [3], and Woodruff-Borden et al. [39] all found no significant relationship between the presence of an anxiety disorder and Full-Scale IQ (FSIQ) in their samples. Einfeld and Tonge [62], however, reported a positive association between anxiety symptoms and FSIQ in individuals with intellectual disabilities, indicating that high rates of anxiety symptoms may be related to higher IQ. It is possible that WS individuals with a higher verbal IQ may be more aware of potential dangers and are able to articulate their negative thoughts and feelings, leading to increased diagnoses [63,64]. To support this notion, though not significant, Dodd & Porter [2] found an association between better verbal abilities (as measured by Woodcock-Johnson Test of Cognitive Ability-Revised; Woodcock & Johnson [65]) and GAD in their sample of WS school-age children and adults. Osório et al. [66], however, found a non-linear pattern of results across age groups, with an association (albeit not significant) between increased verbal IQ and increased Anxious/Depressed and Internalising scores on the Child Behaviour Checklist (CBCL; [67]) in WS children aged 7 to 10 years, and a significant relationship between lower IQ (Full-scale, verbal, and nonverbal) and increased anxiety/depression and internalising behaviours in the adolescent group. Interestingly, Osório et al. also found moderate (non-significant) effect sizes indicating an association between lower FSIQ and nonverbal IQ and increased externalising behaviours in their WS child cohort. Perhaps anxiety symptoms in WS individuals with lower IQ or reduced verbal abilities (i.e., young WS children) may manifest more behaviourally and/or they are not able to articulate their experiences, resulting in less anxiety disorder diagnoses [68]. Given these discrepancies within the current literature pertaining to the relationship between anxiety, age, sex, and IQ, further research is warranted to assist clinicians in determining which young children with WS are most at risk of developing an anxiety disorder and whether this differs depending on the subtype of anxiety.

### 2.2. Longitudinal Studies of Anxiety in WS

There are four longitudinal studies of anxiety in WS individuals known to the author. Firstly, Woodruff-Borden and colleagues [39] examined the longitudinal course (approximately 2 years) of clinically significant levels of anxiety in 4- to 13-year-old WS children and adolescents (*M*age = 6.67 years; *N* = 45). Utilising the Anxiety Disorder Interview Schedule for DSM-IV—Parent Version (ADIS-P; [69]), they found that 27 WS children (60% of sample) presented with at least one anxiety disorder diagnosis at the time of the initial assessment. Consistent with the WS literature (e.g., [1,2,3]), the most common diagnoses were specific phobia (60% ‘loud noises’, 42.22% ‘other’, 40% ‘blood-injury-injection’, 28.89% ‘animals’, and 15.56% ‘natural environment’) and GAD (15.56%). Overall, thirty-seven children (82.2% of the sample) received an anxiety disorder diagnosis during the first phase of the study, with 62.2% of these children continuing to meet diagnostic criteria at follow-up for that same anxiety disorder. Of those children diagnosed with an anxiety disorder during the course of the study, 72.2% also developed an additional anxiety disorder diagnosis, most commonly specific phobia. A reasonable proportion of the sample was being treated pharmacologically (28.9%) or with psychotherapy (17.8%) for their anxiety (although the exact time of treatment was not specified), however, most of these children reportedly continued to experience chronic and debilitating anxiety at follow-up. Overall, these results suggest that clinically significant levels of anxiety are likely to persist over time and seem somewhat resistant to treatment, or at least the current treatment options available for those with WS (in line with [1]). There also appeared to be some variation in the types of anxiety experienced by WS individuals at Time 1 and Time 2, however Woodruff-Borden et al. [39] did not consider environmental factors, such as school changes/transitions or psychosocial stressors, that may also have contributed to the maintenance, type, or development of anxiety over the study time period.

Not all studies indicate that anxiety disorders persist over time in WS. Green et al. [38] assessed anxiety disorders over a period of approximately five-years in WS individuals aged 6 to 23 years (*M*age = 13.1 years; *N* = 25 for follow-up sample). They also included a control group of people with developmental disabilities of mixed aetiology. Using the Kiddies Schedule for Affective Disorders and Schizophrenia for School-Age Children—Present and Lifetime Version (K-SADS-PL; [70]), a semi-structured diagnostic interview based on DSM-IV criteria, Green et al. found a significant decrease in the prevalence rates of anxiety disorders in their WS sample from 21 individuals (84% of sample) at initial assessment to 11 individuals (44%) at follow-up. Two of the five WS individuals (40% of this subset; aged 18- and 20-years) in the sample were being treated with selective serotonin reuptake inhibitors (SSRI) medication over the course of the study (6 years and 1 year duration, respectively) and did not meet the diagnostic criteria for an anxiety disorder under the Diagnostic and Statistical Manual of Mental Disorders, Fourth Edition, Text Revision (DSM-IV-TR; [71]) at follow-up. To explain this result, the authors suggested that, because this percentage decline was similar to the 40% decrease in anxiety for their total WS sample, psychiatric medication did not explain the improvement. Of note, it is unknown if any WS individuals initially diagnosed with an anxiety disorder received psychological treatment. If so, this may explain the decrease in anxiety over time in their sample.

More recently, Ng-Cordell et al. [4] explored anxiety symptomology longitudinally in WS individuals aged 8 to 37 years (*M*age = 19.12 years; *N* = 17). Using the Spence Children’s Anxiety Scale-Parent Version (SCAS-P; [72]) questionnaire, Ng-Cordell et al. reported that many individuals in their sample became more anxious over time (4 years), and, although not statistically significant, effect sizes suggested clinically relevant increases in separation anxiety, obsessive–compulsive disorder, GAD, and social phobia. Fears of physical injury (i.e., specific phobia) reduced from Time 1 to Time 2. There was, however, considerable variability in how anxiety symptomology changed over time in their WS sample, with a small proportion of individuals becoming less anxious over time. Factors contributing to these changes in levels of anxiety symptoms were not explored.

### 2.3. Anxiety in Young Children with WS

There is limited research on anxiety in very young children with WS. To date, only four studies have reported on anxiety in a cohort of children with WS less than 6 years of age and there are only three studies on anxiety in preschool children [41,42,43,44,45,73]. These studies typically utilised general measures of psychopathology and behaviour, such as the CBCL [67,74], rather than a comprehensive measure of anxiety symptomology or a diagnostic screener for anxiety disorders. Of note, the CBCL only looks at broad features of anxiety in six to eight questions (preschool [1.5 to 5 years] and school-age [6 to 18 years] versions, respectively) and does not follow the DSM-5 framework [55]. Nonetheless, available studies suggest that anxiety is also a common issue for young children with WS, although the prevalence rates are mixed.

Using both the preschool and school-age versions of the CBCL, Braga et al. [44] reported that 50% of their WS sample (aged 4 to 6 years; *M*age = 5 years; *N* = 8) fell within either the borderline or clinical range for anxiety problems. In contrast, however, Klein-Tasman and Lee [43] found that approximately 9% of WS children aged 2 to 6 years (*M*age = 4.47 years; *N* = 35) displayed anxiety symptoms within the borderline range using the parent CBCL (parent ratings did not fall in the clinical range) and 14% within the borderline and clinical ranges on the teacher CBCL (3% borderline and 11% clinical). Similarly, Papaeliou et al. [73] and Neo and Tonnsen [45] also found relatively low rates of anxiety-related symptoms using the preschool CBCL [74] in their young WS sample. More specifically, Papaeliou et al. [73] found that no WS child (*M*age = 4.47 years; no age-range available) scored in the borderline or clinical range for the anxiety/depression scale, and Neo and Tonnsen [45] found 11.8% of children (*M*age = 2.58 years; no age-range available) fell in the borderline and clinical range for anxiety problems, combined. These results could indicate that anxiety difficulties are less common in young children with WS. However, the use of the CBCL (a less comprehensive, broad screen of anxiety symptomology [75]) may also account for these findings.

Further research is needed on the prevalence and development of anxiety in young children with WS. As such, the present study employed a longitudinal design to investigate anxiety symptomology in young children with WS and to track their anxiety symptoms over time. Understanding the early trajectory of anxiety in WS will help to: (i) possibly help delineate demographic and environmental contributions to anxiety; and (ii) develop early interventions.

### 2.4. The Current Study

The aims of the current study were three-fold. The first aim was to provide a comprehensive assessment of the prevalence of the different types of anxiety symptoms found in very young children with WS. Symptoms across a wide range of anxiety disorders, as indicated by the Diagnostic and Statistical Manual of Mental Disorders, Fifth Edition (DSM-5; [55]), were investigated. Based on existing literature on older children and adults with WS, and given the biological predisposition to develop an anxiety disorder in WS, it was predicted that the WS children would display more symptoms of anxiety, in particular, symptoms of GAD and specific phobia, than a typically developing comparison group of the same age.

The second aim of the present study was to investigate the developmental course of anxiety in this sample of young WS children over approximately 3.5 years. Although it was unclear from the scant literature available on anxiety in young children with WS, we anticipated high rates of anxiety symptomology in WS, especially in the areas of generalised anxiety and specific phobias, and it was hypothesised that, on average, anxiety symptoms would increase over time (in line with the findings on older children and adults with WS (e.g., [1,4,39]).

The third aim of the current study was to investigate how environmental factors (specifically, anxiety treatment, characteristic medical diagnoses [i.e., hyperacusis and heart disease], significant life events [e.g., changes in the family environment and school transitions]), demographic characteristics [age and sex], and IQ impact on anxiety over time. We expected anxiety to increase alongside a significant environmental change/transition, diagnosis, or significant life stressor. We anticipated some improvement in anxiety symptomology following anxiety treatment, consistent with reports in Kozel et al. [1]. In line with the typically developing preschool children [60], no sex differences were expected in this young WS cohort. We also hypothesised that anxiety would be associated with lower IQ, particularly verbal IQ at this young age.

## 3. Methods

### 3.1. Participants

Nineteen preschool children with a genetically confirmed diagnosis of WS, including 7 males and 12 females, and their parents/guardians participated in this longitudinal study. Genetic testing involved using the Fluorescence in situ hybridization (FISH) test to look for deletion of the elastin gene [17,76,77]. WS children were recruited through Williams Syndrome Australia Limited and the New Zealand Williams Syndrome Association. Early developmental ability for each WS child was assessed using the Mullens Scale of Early Learning (MSEL; [78]). The young WS children performed in the mild to moderate range of disability, on average, with overall developmental quotient (DQ) scores ranging from 28.62 (severe disability) to 68.25 (mild disability) (*M =* 54.64, *SD =* 11.71). The mean DQ scores in the current sample were highly consistent with the means and ranges reported for intellectual levels in the literature for WS, suggesting the present WS cohort was representative [79,80], and the range was consistent with the intellectual and cognitive heterogeneity reported for WS [81,82,83]. Mothers were reported as being their child’s primary caregiver and, on average, completed 14.35 years of education (range 12 to 18 years; *SD* = 1.94). Demographic information regarding each WS family’s ethnic background revealed approximately 63% identified as being Oceanic, 16% as European, with the remainder being Asian, North African, or Middle Eastern. Further demographic data for the WS sample for Time 1 and 2 is shown in Table 1.

### 3.2. Materials

Data on anxiety symptomology (as measured by the Preschool Anxiety Scale [PAS] [85]) and DQ (verbal, nonverbal, and overall ability) were collected at Time 1. The follow-up (Time 2) anxiety data was measured by either the PAS or the Spence Children’s Anxiety Scale-Parent Version (SCAS-P; [72]), depending on the age of the participant at the time and to be consistent with the normative data available. A clinical interview was also completed with each family at Time 1 and Time 2 to assess for specific phobia.

### 3.3. Anxiety Measures

The Preschool Anxiety Scale (PAS; [85]) was designed to assess symptoms of anxiety in preschool children based on the Diagnostic and Statistical Manual of Mental Disorders, Fourth Edition (DSM-IV; [71]). Normative data is currently available for children aged 3 to 5 years. This standardised questionnaire consists of 29 items, with five subscales, each tapping a specific sub-type of anxiety (Generalised Anxiety Disorder, Social Anxiety, Obsessive–Compulsive Disorder, Physical Injury Fears [i.e., specific phobia], and Separation Anxiety). Parents/guardians report on their child’s behaviours related to the five sub-types of anxiety using a 4-point frequency scale ranging from “*Not True at all*” to “*Very Often True*”. The subscales are combined to produce an overall anxiety level (Total Score). The subscales and Total Score yield a *T* score (with population *M* = 50, *SD* = 10). PAS scales are reported to show good to excellent construct validity, cross-informant, and test–retest reliability, and moderate to high internal consistency (see [85,86]).

The Spence Children’s Anxiety Scale-Parent Version (SCAS-P; [72]) is a standardised parent report questionnaire for children aged 6 to 18 years consisting of 38 items and retains the same subscales of anxiety as the PAS, with the addition of a sixth subscale, Panic/Agoraphobia. The SCAS-P has good reliability and validity, of both its factor structure and psychometric properties (see [86,87]).

When interpreting both the PAS and SCAS-P scales, the authors recommend that a score of one standard deviation above the mean for a subscale or Total Score of the community sample should be used as an indicator of clinically elevated anxiety symptoms [85]. This cut-off point for clinical significance has been used in previous WS studies (e.g., [2,4,88]).

Of note, the anxiety scores for the 2- and 3-year-old children in the current WS sample did not significantly differ on any subscale or Total Score for the PAS at Time 1. However, as no 2-year-old norms are available for the Preschool Anxiety Scale [89], we collected a sample of parents/guardian ratings for fourteen typically developing (TD) 2-year-old controls (7 males and 7 females), which were utilised as a community comparison group for the 2-year-old sample of WS children at Time 1 (allowing us to compute *z* scores). Participant details, method of recruitment, demographic data, and descriptive statistics for the 2-year-old TD comparison group are shown in Appendix A.

### 3.4. Combining Anxiety Data for Time 2

For the purposes of this research, and in line with previous studies (see [90]), both the PAS and SCAS-P were utilised depending on the age at Time 2 testing. To increase power for Time 2 analyses, data from the PAS (*n* = 4) and SCAS-P (*n* = 15) were combined and are collectively referred to as ‘anxiety data’. Both anxiety measures are modelled on the same underlying theoretical framework of childhood anxiety, with the PAS utilising several items from the SCAS-P, just reworded so they are appropriate for preschool situations [72,85,91]. As the number of items relating to each subscale differs between the PAS and SCAS-P, *z* scores were used for all Time 1 and Time 2 analyses for ease of comparison.

### 3.5. Developmental Measure

The Mullen Scales of Early Learning (MSEL; [78]) is a standardised measure of early cognitive functioning for infants and preschool children from birth through 68 months (US standardisation sample *N* = 1849). The MSEL consists of four cognitive subtests (Visual Reception, Fine Motor, Receptive Language, and Expressive Language (for further details, see [78]) and a Gross Motor Scale (for children under 33 months). Raw scores on each subtest can be converted to a *T* score (*M* = 50, *SD* = 10). *T* scores for each of the four cognitive subtests are summed to produce the Early Learning Composite standard score (*M* = 100, *SD* = 15). Comparable to an intelligence quotient, the Early Learning Composite score resembles an overall developmental quotient, where a lower Early Learning Composite score indicates reduced cognitive ability. The MSEL has adequate psychometric properties. It was reported that the median internal consistency values across each age group ranges from 0.75 to 0.83 for the five subtests [78]. Across all ages: the internal consistency of the Early Learning Composite is high, ranging from 0.83 to 0.95 (median value was 0.91); test–retest reliability correlations range from 0.71 to 0.96; and interscorer reliability range from 0.91 to 0.99. In line with other published studies with young children with neurodevelopmental disorders, including WS (e.g., [90,92]), DQ scores were utilised as some of the young WS children were at floor (standard *T* score of 20) on each of the individual MSEL cognitive subtests. DQ scores were calculated for each MSEL subtest utilising the formula: DQ = age equivalent scores/chronological age × 100. Verbal DQ was computed by averaging the DQ scores across the Receptive and Expressive MSEL subtests, Nonverbal DQ by averaging across the MSEL Visual Reception and Fine Motor, and the four subtest DQs were then averaged to create an overall DQ. As such, the three DQ scores (i.e., Global, Verbal, and Nonverbal) were used for the analyses as a measure of each child’s level of development.

### 3.6. Analytic Approach

This research was part of a wider research study. Ethics approval for this study was gained from the Macquarie University Human Research Ethics Committee (reference number: 5200900071 and 52021913524613). Details of the research were then sent to the Williams Syndrome Australia Limited and the New Zealand Williams Syndrome Association, who, in turn, forwarded the information onto their members. Investigators were contacted directly by families who were interested in participating. Written consent was obtained from all parents/guardians. The MSEL was administered by an experienced researcher (first author and neuropsychologist in training) who received appropriate training to administer the measure according to the instructions in the examiner’s manual (see [78]). The order of MSEL items were randomised to avoid any systematic effects on tasks and to maintain children’s motivation, as per the standardised instructions [78]. The MSEL Gross Motor subtest was not administered as some children in the current sample were outside the age range. A clinical interview was also conducted by a trained clinician (first author) with each parent/guardian to obtain information pertaining to demographics and symptoms of social phobia (based on DSM-5 criteria). The SCAS-P was administered to parents with children aged 6 years and over (*n* = 15) at Time 2, and the SCAS-P Panic/Agoraphobia sub-type was excluded in the analyses for this study. Note, all data was collected prior to the COVID-19 pandemic and, as such, findings of the current study were not affected by any anxiety which may have occurred as a direct result of any environmental, situational, or medical interventions associated with this virus.

Corrections for multiple comparisons were not applied due to low power. This approach is in line with other published studies on WS (e.g., [64,93,94,95]). To minimise the likelihood of a Type II error, the *p* value was set to 0.05 for all analyses (see [96]). For any coefficients at or near the 0.05 level of significance, moderate to large effect sizes would be reported to assist with interpretation and support the decision to take this approach, demonstrating that the findings are not simply a reflection of Type I error [96]. Classification of effect sizes for *r* are as follows: ≤0.1 = small; 0.3 = medium; and ≥0.5 = large; classification of effect sizes for *d* are as follows: ≤0.2 = small; 0.5 = medium; and ≥0.8 = large [97]. Two anxiety scales were found to violate assumptions, and, where appropriate, nonparametric tests were engaged to check the robustness of the result.

## 4. Results

### 4.1. Time 1—Prevalence of Anxiety in Young Children with WS

Anxiety profiles (*z* scores) and specific phobias (percentage of total sample) for Time 1 (as measured by the PAS) and Time 2 (as measured by both the PAS and SCAS-P) are presented in Table 2.

Using the cut-off score of one standard deviation (*SD*) above the ‘total’ group mean of the community sample (in line with [98]), we calculated the percentage of young children with WS in our sample that displayed clinically elevated scores for each anxiety subtype and Total Score at Time 1 and Time 2 (see Table 2). At Time 1, nine young WS children (47.37% of the total sample) were reported to experience at least one clinically elevated anxiety subscale on the PAS, showing a high rate of anxiety symptomology in this very young sample. In contrast, at Time 2, 16 WS children (84.21% of the total sample) displayed at least one clinically elevated anxiety symptom. The most common area of symptom difficulty in young children with WS at Time 1 was Generalised Anxiety Disorder (36.84% of total sample), followed by Physical Injury Fears (15.79%), Separation Anxiety (10.53%), and then Obsessive–Compulsive Disorder (5.26%). No young children with WS in the current sample were reported to experience clinically elevated social anxiety symptoms at Time 1. Furthermore, no WS child fell into the clinically elevated range for the PAS Total Score. In contrast, however, the most common area of symptom difficulty at Time 2 was Physical Injury Fears (52.63% of the total sample), followed by Generalised Anxiety Disorder (47.37%). Furthermore, 31.58% of the total sample fell in the clinically elevated range for the anxiety Total Score at Time 2 (0% at Time 1). In line with Time 1, on average, no young children with WS were reported to experience clinically elevated social anxiety symptoms at follow-up. There was also an increase in comorbidity rates at Time 2, with three (15.79% of total sample) WS children at Time 1, and 10 (52.63%) at Time 2, falling into the clinically elevated range on more than one anxiety subtype.

Based on clinical interview, 12 (63.16% of total sample) young children in our cohort met the DSM-5 diagnostic criteria for specific phobia at Time 1. Of note, to meet criteria, a child was required to display both: (i) the symptoms of specific phobia, and (ii) their fear or anxiety had to cause a significant functional impact (for example, a vacuum cleaner can never be used while the child is in the room). Of those 12, 58.33% reported phobia of loud noises, 41.67% for situational phobia (for example, new/unexpected situations, bath-time, or crowded places), 25.00% fell within the ‘other’ category for specific phobia (for example, steps, a change in colour on the ground, new toys, or equipment), 16.67% for blood-injury-injection phobia, and 8.33% for both natural environment phobia and phobia of animals. Furthermore, six of these children met the DSM-5 diagnostic criteria for more than one specific phobia subtype at Time 1. In contrast, overall, the percentage of WS children meeting DSM-5 criteria for each specific phobia subtype increased at Time 2, apart from the ‘other’ specific phobia category (which decreased from 15.79% to 10.53%). Sixteen (84.21% of total sample) WS children met the DSM-5 diagnostic criteria for specific phobia at follow-up testing. Of these 16 children, and in line with the Time 1 group profile, the most common phobia continued to be loud noises (62.50%), followed by the situational phobia (56.25%). However, in contrast to Time 1, the blood-injury-injection phobia and phobia of animals (both with 31.25%) were the third most common phobia subtypes in this young WS cohort at Time 2, followed by natural environment phobia (25%), and then the ‘other’ specific phobia category (12.50%). There was also an increase in comorbidity rates, with ten WS children meeting the DSM-5 diagnostic criteria for more than one specific phobia subtype at Time 2.

### 4.2. Time 2—Longitudinal Trajectory of Anxiety in Young Children with WS

**Figure 1:**
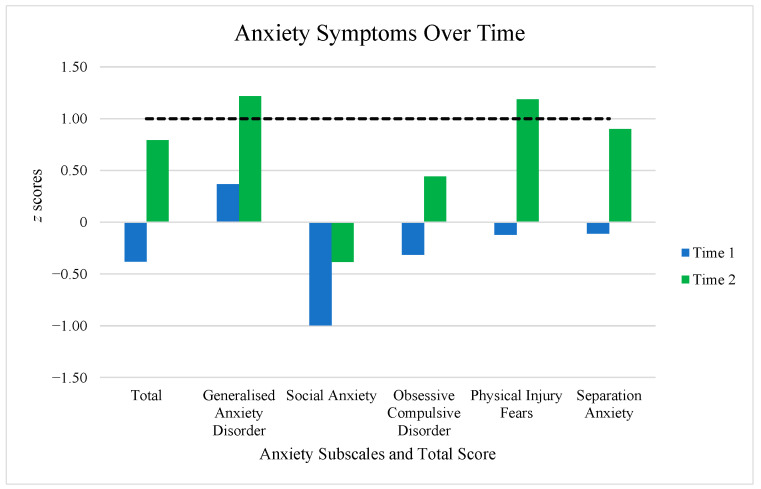
Longitudinal anxiety data (PAS *z* scores for Time 1 and combined PAS and SCAS-P *z* scores for Time 2) for each anxiety subtype and the Total Anxiety Score by WS child (*N* = 19). Figure 1 illustrates the average change in anxiety profiles across young children with WS over time. The dotted line indicates the clinical cut-off of one standard deviation above the community sample mean.

The mean difference in age for the WS children between Time 1 and Time 2 was 3.45 years (*SD* = 1.55). Table 2 shows the change in anxiety scores over time. The mean *z* scores increased for all anxiety subtypes and Total Score over time. Paired-samples t-tests confirmed that the change in all mean *z* scores from Time 1 to Time 2 were significant (all *p* values < 0.02). Figure 1 further illustrates the changes in anxiety over time for this very young WS cohort. Of note, both Generalised Anxiety Disorder and Physical Injury Fears mean *z* scores at Time 2 increased from outside the clinical range to clinically elevated levels, on average. The percentages of young WS children falling in the clinical range also increased for all anxiety subtypes and Total Score over time, apart from social anxiety which remained at 0% of the total sample.

Table 3 displays the profile of anxiety symptomology (i.e., clinically elevated anxiety subtypes and/or specific phobia diagnosis), environmental factors, additional diagnoses, and psychological intervention for each WS child in the current sample. What was evident was the prevalence of anxiety across Time 1 and Time 2, and, qualitatively, the probability of a young WS child developing additional anxiety symptoms or phobias, appears very high. Of those fourteen WS children with a clinically elevated anxiety symptom or specific phobia diagnosis at Time 1, thirteen (68.42% of total sample) developed additional and/or different anxiety difficulties by their Time 2 assessment. The five WS children who did not display any significant symptoms at Time 1 had all developed significant elevated levels of anxiety symptoms and/or a specific phobia diagnosis by their Time 2 follow-up.

### 4.3. Heterogeneity of Anxiety in Young Children with WS over Time

There was considerable individual variability within each of the anxiety/specific phobia subtypes and the total anxiety score (see Table 2 and Table 3). In terms of anxiety, although most young WS children displayed an increase in anxiety for all anxiety subtypes and the Total Score, there was a proportion of children who experienced a reduction in anxiety symptomology over time (see Appendix B for graphical representation of the diversity in anxiety score for this young WS cohort). Furthermore, apart from social anxiety (see Table 2), anxiety levels generally increased over time with a considerable number of WS children displaying clinically elevated scores at Time 2. Of those children who became more anxious over time (across all anxiety subtypes and Total Score), 16 displayed an increase in more than 1 *SD* based on the normative sample (indicating a reliable change), 10 of whom displayed a reliable increase on more than one anxiety subtype. Four WS children displayed a reliable decrease in anxiety (i.e., reduction in more than 1 *SD*). More specifically, one child on Physical Injury Fears; one on Generalised Anxiety Disorder; one on Obsessive–Compulsive Disorder; and another on both Generalised Anxiety Disorder and Separation Anxiety. Three WS children displayed no reliable change in any anxiety symptomology over time.

**Figure 2:**
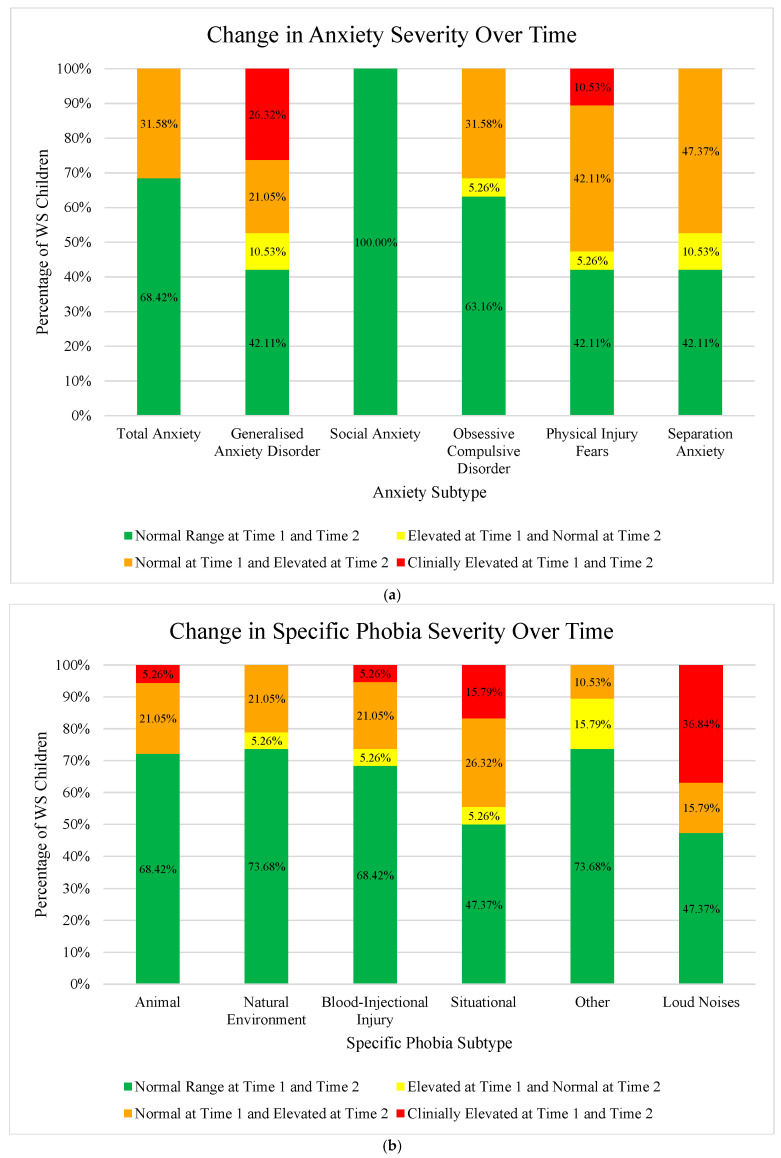
The percentage of the total WS children in the current sample who fell into each category of clinically elevated anxiety symptoms (**a**) and specific phobia diagnosis (**b**) at Time 1 and Time 2. Figure 2 illustrates the vast heterogeneity in anxiety profiles across young children with WS. The categories consisted of: (1) falling within the normal range at Time 1 and Time 2 (green); (2) clinically elevated at Time 1 to normal range at Time 2 (i.e., decrease in anxiety; yellow); (3) normal range at Time 1 to clinical range at Time 2 (i.e., increase in anxiety over time; orange); and (4) clinically elevated at Time 1 and Time 2 (red).

To further investigate individual changes in each anxiety subtype, Total Score, and specific phobia diagnosis over time for this WS cohort (i.e., whether a child fell within the normal range or displayed clinically elevated anxiety scores at each time point) stacked column charts were created (see Figure 2). The categories consisted of: (1) falling within the normal range at Time 1 and Time 2 (green); (2) clinically elevated range at Time 1 to normal range at Time 2 (i.e., decrease in anxiety over time; yellow); (3) normal range at Time 1 to clinically elevated range at Time 2 (i.e., increase in anxiety at Time 2; orange); and (4) clinically elevated levels of anxiety at Time 1 and Time 2 (red). The highest proportion of WS children fell within the normal range at Time 1 and Time 2 for each anxiety subtype, Total Score, and specific phobia, apart from Separation Anxiety which displayed the highest percentage of children reaching clinical levels at Time 2 (47.37% of total sample). Physical Injury Fears (42.11%), and Obsessive–Compulsive Disorder and the Total Score (both at 31.58%) also saw a considerable number of WS children reaching clinical significance at Time 2 (Figure 2a). A considerable proportion of WS children received a diagnosis of Situational (26.32%), Animal (21.05%), Natural Environment (21.05%), and Blood-Injectional Injury (21.05%) specific phobias at Time 2 (Figure 2b). Generalised Anxiety Disorder (Figure 2a) and specific phobia of loud noises (Figure 2b) also contained a substantial proportion of WS children who displayed clinically elevated levels at both time points (36.84% and 26.32%, respectively). Generally, most WS children did not reduce levels of anxiety/specific phobia symptomology over time; the ‘other’ specific phobia (Figure 2b) was the only subtype which displayed a higher proportion of WS children with reduced clinically elevated symptoms (in comparison to increasing symptoms) at Time 2.

Table 3, which displays the diagnostic profile of anxiety symptomology for each WS child in the current sample, also highlights the considerable heterogeneity of anxiety symptoms and specific phobias experienced by each child and the difference in when any anxiety arose (Time 1 or Time 2).

### 4.4. Environmental Contributions to Anxiety Change over Time

Environmental influences and their contribution to anxiety symptomology and specific phobia diagnoses in very young children with WS over time was also examined (see Table 3 for a detailed list of significant life events and treatment for anxiety experienced by each WS child). A ‘significant life event’ was defined as a specific experience or transition that alters the regular flow of an individual’s life [99,100]. A significant life event was either *normative* (i.e., beginning day-care/primary school and the birth of a sibling) or *non-normative* (i.e., parental separation or undergoing surgery). Inter-rater reliability was undertaken to confirm these classifications. Of note, one child was diagnosed with Attention-Deficit/Hyperactivity Disorder (ADHD) between Time 1 and Time 2. No children were medicated for anxiety, low mood, or ADHD during the length of the study. Three WS children (15.79%) were receiving psychological intervention between Time 1 and Time 2 testing: one child was receiving psychological intervention for generalised anxiety and aggression; one child was receiving psychotherapy intervention for anxiety; and one child was receiving psychological intervention for low mood. Of the two children (10.53% of the total sample) that were receiving psychotherapy intervention for anxiety between Time 1 and Time 2, both continued to display clinically elevated anxiety symptomology at Time 2 (with raw scores increasing at Time 2).

Fifteen children (78.95%) experienced a significant life event (10 normative and five non-normative) prior to Time 1 testing. Between Time 1 and Time 2 testing, 13 children (68.42%) experienced a significant life event (11 normative and two non-normative) Independent samples t-tests indicated that, on average, anxiety scores did not significantly change between WS children who experienced a significant life event and those who did not (all *p* values > 0.05).

Eighteen (94.74% of the current sample) WS children in the sample were diagnosed with hyperacusis and sixteen (84.21%) were diagnosed with heart problems. In line with our hypotheses, a series of 1-tailed Pearson correlation coefficients were performed to assess the relationship between WS children diagnosed with either cardiovascular abnormalities or hyperacusis and those that met the DSM-5 diagnostic criteria for a specific phobia of blood- injectional injury or loud noises, respectively.

#### 4.4.1. Relationship Between Cardiovascular Abnormalities and Symptoms of a Phobia of Blood Injectional Injury

At Time 1, there was a medium effect size between children with heart problems and meeting diagnostic criteria for a specific phobia of blood injectional injury at Time 1 testing (*r*_b_ (19) = 0.32, *p* (one tailed) = 0.090). However, a significant association was reached at the Time 2 follow-up testing (*r*_b_ (19) = 0.40, *p* (one tailed) = 0.046). Overall, the results indicate that very young WS children who have cardiovascular abnormalities are more likely to meet diagnostic criteria for a specific phobia of blood injectional injury.

#### 4.4.2. Relationship Between Hyperacusis and Symptoms of a Phobia of Loud Noises

No significant associations were found between WS children who had been diagnosed with hyperacusis and meeting diagnostic criteria for a specific phobia of loud noises at either time points (all *p* values > 0.05).

#### 4.4.3. Relationship Between Anxiety and Chronological Age, Sex, and DQ over Time

A series of Pearson and Spearman’s Rho correlation coefficients were performed to assess the relationship between chronological age, sex, and DQ score (verbal, nonverbal, and global) and the change in anxiety (calculated as the difference between the corresponding Time 1 PAS and Time 2 combined PAS/SCAS-P *z* scores). All correlations are reported in Table 4.

#### 4.4.4. Relationship Between Chronological Age and Anxiety over Time

**Figure A1:**
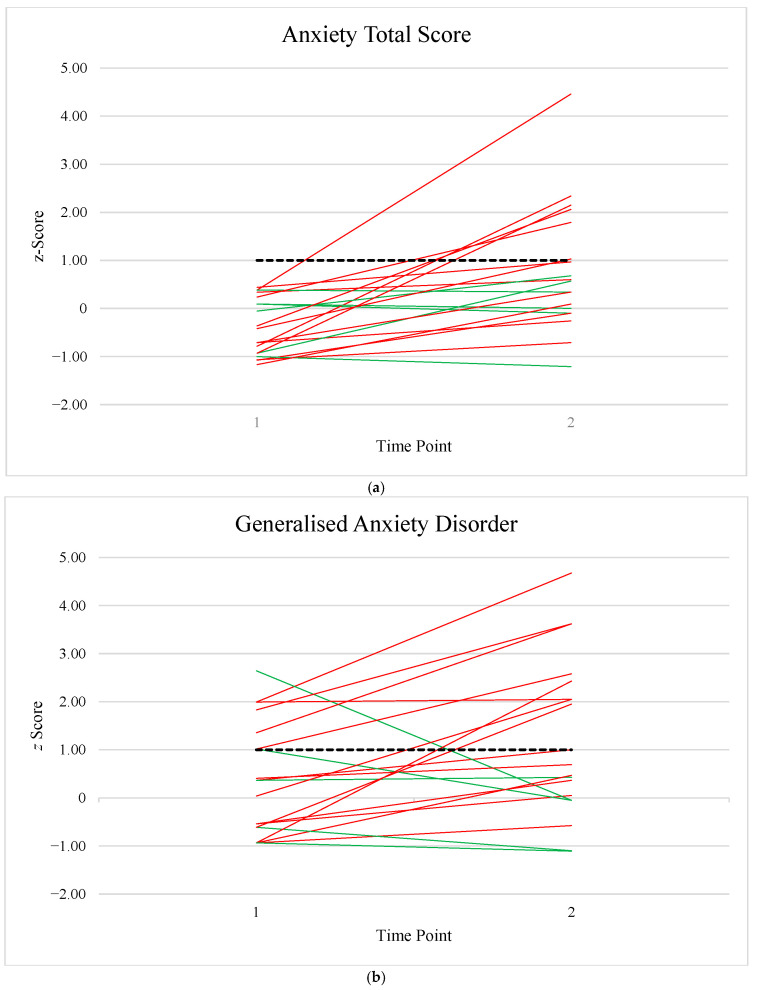

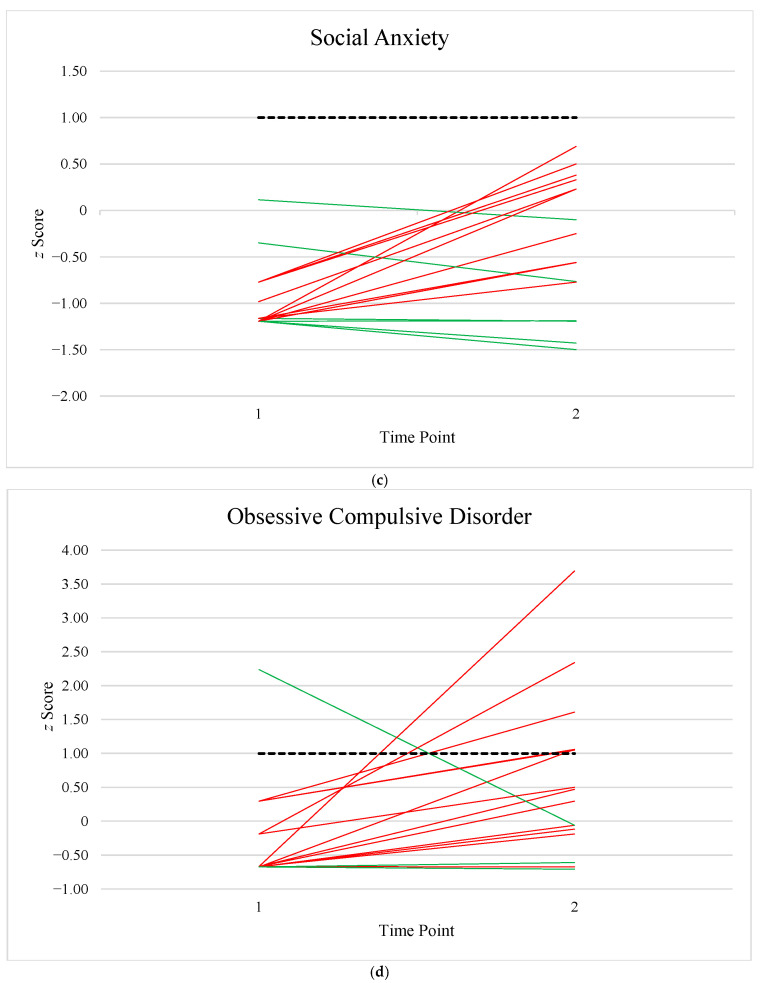

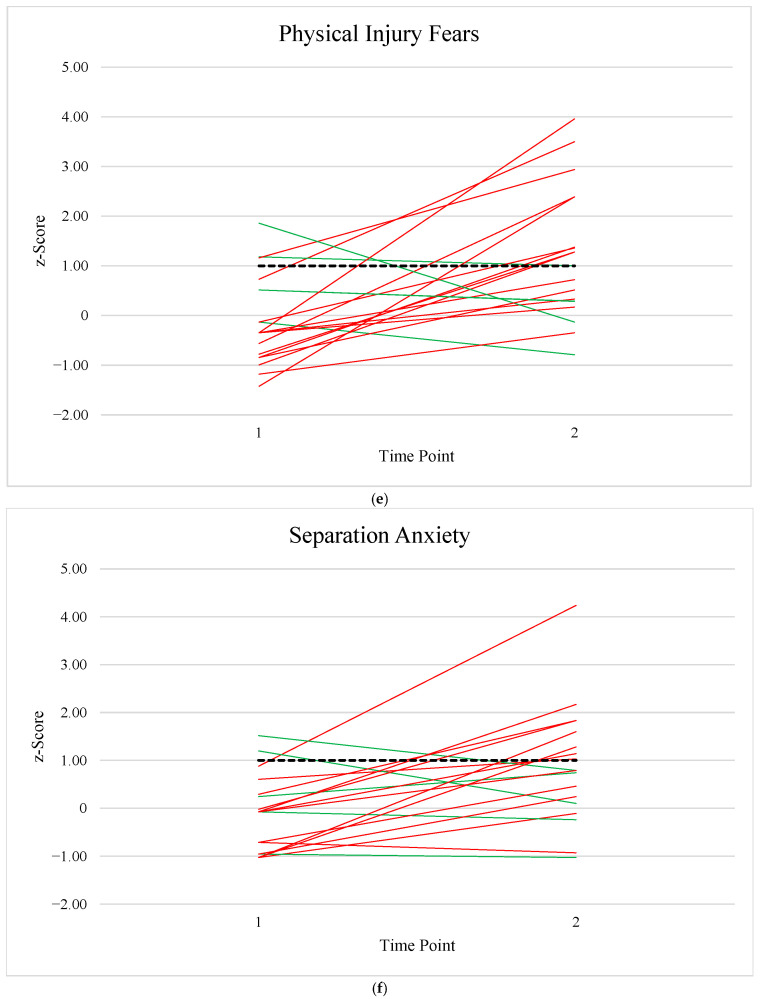
Longitudinal anxiety data (PAS *z* scores for Time 1 and combined PAS and SCAS-P *z* scores for Time 2) for each anxiety subtype and the Total Score by WS child. Lines in red display an increase in anxiety *z* scores from time 1 to time 2 and the lines in green display a decrease in anxiety. The dotted line indicates the clinical cut-off of 1 *SD* above the community sample mean.

Chronological age was significantly and positively correlated with social anxiety (*r* (19) = 0.50, *p* = 0.032). A medium positive effect size was also found between age and Physical Injury Fears (*r* (19) = 0.31, *p* = 0.194) and Separation Anxiety (*r* (19) = 0.355, *p* = 0.136). These results indicate that symptoms of social anxiety, separation anxiety, and specific fears increase with age in young children with WS. In relation to social anxiety symptomology, unlike for separation anxiety, and specific fears/phobias, however, no child met the clinical cut-off or clinical levels of social anxiety, consistent with a likely diagnosis of social anxiety disorder (See Figure A1).

#### 4.4.5. Relationship Between Sex and Anxiety over Time

Sex was significantly and positively correlated with Physical Injury Fears (*r_pb_* (19) = 0.61, *p* = 0.005), indicating that, on average, female WS children display increased symptoms of specific fears over time. In contrast, a negative medium effect size was found between sex and Generalised Anxiety Disorder (*r_pb_* (19) = −0.41, *p* = 0.083), suggesting that males display more symptoms over time.

#### 4.4.6. Relationship Between Verbal, Nonverbal, and Global DQ and Anxiety over Time

Although no significant relationships were identified, effect sizes suggested clinically relevant associations between lower Verbal DQ and increased symptoms of Generalised Anxiety Disorder (*r* (19) = −0.34, *p* = 0.153) and the anxiety Total Score (*r_s_* (19) = −0.34, *p* = 0.156). A medium, positive effect size was also found between Overall DQ and Obsessive–Compulsive Disorder (*r_s_* (19) = 0.30, *p* = 0.205), indicating a higher overall IQ is associated with increased obsessive compulsive symptomology over time.

## 5. Discussion

Despite well documented issues with anxiety in WS, the early trajectory of anxiety is not well understood. This is, to the best of our knowledge, the first study to investigate the longitudinal profile of anxiety symptomology in very young children with WS. Anxiety profiles were examined at both group and individual levels. Potential environmental and demographic correlates of anxiety symptomology were also explored.

### 5.1. Prevalence of Anxiety in Very Young Children with WS

The first aim of the study was to provide a comprehensive estimate of the prevalence of the different types of anxiety symptoms found in very young children with WS. Overall, and in line with previous studies on older children and adults with WS [4], almost three-quarters of the current sample experienced at least one clinically elevated anxiety symptom at Time 1. This result was much higher than the previously reported prevalence of anxiety in preschool children with WS [41,42,43,44,45,73], albeit based on less-than-optimal, broad screening measures of anxiety, such as the CBCL. Use of a comprehensive anxiety questionnaire based on DSM-5 criteria in the present study most likely provided a more accurate reflection of the prevalence of anxiety in this very young WS group and is more consistent with the prevalence rates reported for WS preschoolers in a recent comprehensive clinical description of WS [1]. The anxiety rates found in the current study were also higher than the estimated 12% prevalence in children with ID, and the estimated global rate of 7–11% reported for typically developing children [20,101,102]. As such, this key finding further supports the notion that anxiety is highly prevalent in WS individuals and, more specifically, provides evidence of high rates of anxiety symptoms from a very young age.

As predicted, and consistent with previous research [3,20], the current young WS sample displayed particularly high levels of specific phobia and GAD, both at Time 1 and at Time 2. Furthermore, no clinically elevated levels of social phobia were found, which is consistent with studies on older children and adults with WS (e.g., [38]). In line with the previous literature (e.g., [3,38,39]), the most common specific phobias related to loud noises and particular situations (most commonly, crowded places and new/unexpected situations), which is consistent with the high rates of hyperacusis and, perhaps, visits to unfamiliar medical professionals (often children will encounter medical consultations at this young age). In contrast with the studies of older children and adults with WS, however, only 16% of young WS children met criteria for a blood-injury-injection-specific phobia at Time 1—this rate increased dramatically at Time 2, with almost one third of our WS cohort meeting criteria at this time point; this is likely a reflection of the ongoing hospitalisations and medical interventions experienced by those WS children [21]. Contrary to Kozel et al. [1], who reported no prevalence of obsessive–compulsive disorder in WS preschool children aged 3 to 5 years (an estimate based on a lack of research), we found that 5% of our sample fell in the clinical range for obsessive–compulsive disorder at Time 1, which then increased to over 30% of the young WS sample at Time 2.

Additionally, consistent with the clinical variability previously reported in the WS literature (e.g., [80,82,83]), and in line with developmental models such as the bio-psycho-social model proposed by Dennis et al. [103], whereby biological, cognitive, and environmental events are all considered to impact an individual’s cognitive phenotype, there was considerable individual variability within each of the anxiety/specific phobia subtypes and the total anxiety score at Time 1 and Time 2.

### 5.2. Developmental Trajectory of Anxiety in Young WS Children

In contrast to community samples where anxiety prevalence estimates remain relatively stable over development [29], the mean *z* scores and percentages of young WS children falling in the clinical range significantly increased across all anxiety subtypes and for the total anxiety score over time in the current study. In line with our hypothesis, and consistent with prior longitudinal WS research [4,39], our results indicated that WS children became more anxious over time, even at this very young age, perhaps reflecting a combination of their genetic/biological predisposition for developing anxiety, as well as the increased likelihood of environmental contributions/triggers for anxiety with time. A large proportion of WS children (78.57% of the total follow-up sample) also developed symptoms of an additional anxiety disorder over the course of the study, consistent with Woodruff-Borden and colleagues [39]. Of note, however, there was considerable individual variability in terms of how anxiety changed over time in this young WS sample, with some individuals showing reduced anxiety over time, the opposite to the general trends observed in WS and outlined above. These results highlight that it is essential for future studies to systematically investigate the potential risk and protective factors that may be associated with these longitudinal changes in anxiety and this, indeed, may hold the key to successful preventions and/or anxiety interventions for the WS population.

### 5.3. Environmental Influences on Anxiety in Young WS Children

Another unique aspect of the current study was the investigation into select environmental changes that contributed to anxiety symptomology. In contrast to our predictions, in the current study using parent report measures, anxiety symptomology in young WS children did not change over time as a reflection of environmental change (e.g., a significant life event). Moreover, anxiety symptomology did not appear to change significantly with the implementation of anxiety interventions. For example, WS children receiving psychological therapy intervention for anxiety between Time 1 and Time 2 continued to display clinically elevated anxiety symptomology at Time 2. Unfortunately, as it was beyond the scope of the current study, the length or types of anxiety treatment these children were receiving are unknown, however future studies should closely examine treatment outcomes for individuals with WS [1]. For typically developing preschool children, psychotherapy is the first line treatment for anxiety disorders, commonly age-adjusted cognitive-behavioural therapy [104], however anxiety intervention for young children is generally a combination of psychoeducation for parents, family training, and play therapy [105]. Psychopharmacological treatments are not currently recommended as a first line treatment for anxiety in young children under the age of 6 years, at least for TD children, as they have not yet been empirically tested [106,107]. Future studies looking at the efficacy of various anxiety treatments in WS is clearly warranted, with some promise of modifications to cognitive behaviour therapy as a treatment of anxiety in WS and those with intellectual disability [1,108].

In line with our predictions, although not significant at Time 1, these preliminary findings found a moderate effect size indicating an association between very young WS children with cardiovascular abnormalities and those meeting diagnostic criteria for a specific phobia of blood injectional injury. This finding was further evidenced at Time 2 (significant association on this occasion), perhaps reflecting repeated treatments and interventions for heart conditions, which may also be impacting on the levels of specific phobia of blood injectional injury. What we did not expect, however, was a failure to find an association between young WS children who had been diagnosed with hyperacusis and meeting diagnostic criteria for a specific phobia of loud noises (at Time 1 or Time 2). This finding was in contrast to findings from Uljarević et al. [40], who reported a significant association between sensory hypersensitivity and increased levels of anxiety in adolescents and adults with WS. Perhaps a more formal measure of hyperacusis is required, as the present study simply relied on a parental clinical interview for diagnosis and severity. Moreover, sensory sensitivity is not necessarily related to hyperacusis per se, and sensory questionnaire measures, along with hyperacusis measures, would be good to include within the one study to further tease these variables apart.

### 5.4. The Association Between Anxiety over Time and Chronological Age, Sex, and IQ

Another aim of this study was to investigate the role of demographic factors in the development and chronicity of anxiety symptomology in very young children with WS, as findings are mixed in the literature. A significant and positive association was found between chronological age and social anxiety and, although not significant, effect sizes also indicated a similar trend for separation anxiety in line with Ng-Cordell et al. [4]. Unlike Ng-Cordell et al., however, the present study also found that chronological age was positively associated with increased fears of physical injury. These results provide initial support for age-related contributions to anxiety in WS (in contrast with findings for typically developing preschoolers; [29]). However, the relationship between chronological age and anxiety may be non-linear, as suggested by Dodd and Porter [2] and Green et al. [38], and may perhaps reflect critical periods during development where certain anxiety symptoms may be more likely to occur, for some reason, in WS individuals. For example, during periods of transition and/or change, like starting preschool or school.

In contrast to our prediction of no sex differences, the present study indicated that young WS females showed increased levels of physical injury fears over time. This finding is also consistent with those of previous studies on older children and adults with WS, which suggested that specific phobias are more prevalent in females [2,21]. Further, moderate effect sizes indicated that males displayed more generalised anxiety symptoms than females over time, a pattern that is different to the pattern reported in the TD population [109].

In line with predictions, although not significant, moderate effect sizes in the present study indicated a relationship between lower verbal DQ and heightened symptoms of GAD and total anxiety over time. A nonsignificant association was also indicated for higher overall DQ and increased obsessive compulsive symptomology over time (moderate effect size). Taken together with previous research on older children, adolescents, and adults with WS [2,66], this finding perhaps reflects a change in the manifestation of anxiety over time. For example, lower verbal DQ in young children with WS may be associated with reduced expressive language abilities, so they are not able to verbalise their feelings of anxiety and, as such, anxiety may manifest more behaviourally, masking the typical symptoms of anxiety which results in less anxiety diagnoses at this young age [68]. In contrast, older children, adolescents, and adults with WS generally have better developed expressive language abilities and can verbalise their feelings, articulate their experiences, and, perhaps, are better able to articulate their anxious thoughts. Also of note, as with age and anxiety, the relationship between intellect and anxiety may not be linear. Overall, these findings suggest that anxiety in WS may manifest differently according to age, sex, and ability levels, and this warrants further longitudinal investigation with larger samples over multiple time periods. Examination of anxious thoughts and experiences through interview with those with WS, and their families, is also warranted to better understand anxiety in WS (e.g., see [108]).

### 5.5. Considerations and Future Research

The literature on anxiety in WS, whilst abundant relative to other topics, is still in its infancy, with a lot more research required in order to understand the biological and environmental contributions towards anxiety manifestations in WS, this might include, in future studies, parent–child dynamics and child temperament (e.g., behavioural inhibition). Large, trans-disciplinary studies are required in order to progress this field further, preferably with gold standard measures of anxiety, including clinical interviews and diagnostic measures conducted by trained clinicians. More research on anxiety treatment efficacy is also warranted across different age groups.

Although the present study was the first of its kind in terms of its longitudinal focus in young persons with WS and its exploration of the role of environmental contributions towards the trajectory of anxiety in WS, several limitations must be considered when interpreting the findings and should be addressed in future research. Firstly, although our sample size (*N* = 19) is consistent with sample sizes of WS research to date, the current study had limited statistical power to detect significant effects due to this small sample size. While significance levels and effect sizes are usually indicative of adequate power, and have been reported in the current study, a larger sample size would allow for more generalisability and robust results and would also minimise sampling errors. Because WS is a rare syndrome, however, it is very difficult to recruit large numbers, particularly for very restricted age ranges (unless recruitment is international). Even so, the present study spanned across Australia and New Zealand in order to recruit larger samples. Larger studies of young WS children would also allow for clarification of the associations between the development of anxiety and individual experiences and demographic characteristics (e.g., sex and IQ), as well as cultural influences if recruitment spanned across a number of diverse countries, thus further increasing generalisability of findings. Similarly, in-depth research on the association between hyperacusis and the development of a fear of loud noises should be undertaken with more appropriate tests utilised to measure sound-sensitivity, rather than basing this on parent report, and perhaps involving cross-syndrome comparisons with other neurodevelopmental conditions known to be associated with hyperacusis, such as autism spectrum disorder [110]. Studies examining comorbidities known to be associated with WS and anxiety and that are, in themselves, particularly common in WS, are also warranted, such as the developmental trajectory of anxiety in those with WS who also have co-existing ADHD, Autism Spectrum Disorder (ASD), and/or sensory processing disorder. These coexisting conditions might increase the likelihood of anxiety in those with WS, or may, perhaps, exacerbate the functional impacts.

Future research should also focus on examining the effectiveness of current anxiety treatments in young children with WS, for example, implementing therapeutic trials to identify appropriate treatment interventions for this population, including not only pharmacological treatments, but also psychological interventions such as modified cognitive behaviour therapy [1,108]. Further, research with typically developing populations suggests that parent involvement in anxiety treatment is beneficial for young children [111], and this should also be trialled in the WS population. Preventative interventions are also warranted in WS, given the unique opportunity here with the known biological predisposition towards anxiety in WS.

Another issue concerns the utilisation of a screening measure to identify the prevalence of anxiety subtypes (i.e., PAS and SCAS-P). Screening measures are rarely validated for individuals with ID or neurodevelopmental disorders, and we acknowledge that an individual’s internal anxiety symptomology may present differently in WS, including behavioural manifestations outside the norm of what is seen in neurotypical individuals with anxiety [112]. Future research should attempt to develop a more sensitive and valid measure of anxiety for these atypical populations, including items tailored to what we know about anxiety and its various manifestations in ID and in WS. Unless a lifelong diagnostic interview is utilised, screening measures also only measure anxiety symptoms over a single time point and, as such, may not fully capture the extent of the anxiety symptomology experienced by the individual.

Additionally, the present study relied on parent insights, utilising parent report ratings of anxiety, which may lead to retrospective or subject bias (for further details see [21]). As self-report measures are not appropriate for this young age group, and are perhaps not optimal for those with intellectual disability [113,114], research should aim to incorporate information from multiple sources (i.e., day-care/preschool or schoolteachers, and their caregivers), and clinical interviews and observations with multiple informants. We also acknowledge the clinical interview utilised to provide a diagnosis of specific phobia for the current study was not standardised. However, as the parent interviews were carried out by a trained clinician (psychologist in training, first author), under the supervision of a senior clinical neuropsychologist (second author), and were based on DSM-5 criteria, this is likely a true reflection of the prevalence rates of specific phobia symptomology in WS.

Although it was beyond the scope of the current study, future research should also examine the associations between the development of anxiety in very young children with WS and their family history of anxiety and other mental health disorders, as we know that anxiety is highly heritable, and having an anxious parent also increases the likelihood of a child developing anxiety [115]. Despite WS itself being a biological risk factor for anxiety, these other heritable contributions should not be overlooked. Furthermore, family context and home environment should also be investigated as potential contributing factors, as these are known to relate to anxiety in neurotypical children [116] and are easy targets for intervention. Finally, continued longitudinal research is essential to gain a detailed understanding of the developmental trajectory of anxiety in WS individuals, and, ideally, utilising neuropsychological, environmental, and biological measures to investigate other potential contributions outside of those reported in the current study, such as more specific cognitive abilities and hormonal contributions. Some of these factors may be protective, such as the high levels of oxytocin reported in WS [117] being a likely protective factor against social anxiety, and some may exacerbate anxiety, such as cognitive flexibility issues reported in WS (e.g., [3,95]), and may make it more likely that WS individuals become stuck on their anxious thoughts and may be a potential barrier to anxiety treatments such as cognitive behavioural therapy. Furthermore, due to our small sample size, we were limited with the types of analyses we were able to compute when investigating significant life events, and as such, we were not able to delineate the impact between the different types of events and how they contribute to the development and/or maintenance of anxiety in WS children. Again, this is an important area of research that should be addressed in future studies.

### 5.6. Clinical Implications

The high prevalence of anxiety among very young children with WS reveals the importance of screening this population at a very early age. As early interventions greatly improve an individual’s prognosis [118], and given the evidenced early onset and chronic course of anxiety in young children with WS, early identification and intervention seems especially important in this population using valid and reliable measures to identify each individual’s specific anxiety disorder profile.

Clinicians and parents/caregivers should also be aware of the clinical presentation of anxiety in young children with WS (or other intellectual disabilities), which may differ from older children and adults with WS and their typically developing peers. For example, due to their reduced verbal abilities, externalising/challenging behaviours may be a symptom of anxiety at this young age group. As such, the existing anxiety classification systems may be missing important symptoms/behaviours for this age group and should be reviewed to reflect specific symptomology in young children with intellectual disabilities or delayed verbal skills. Repetitive thoughts and behaviours (including stereotypic behaviours and obsessions/compulsions) are also common in WS individuals, and increased severity of obsessions has been found to be associated with increased levels of anxiety [119].

Lastly, the findings in the current study provide important insights into the relationship between risk factors and anxiety in young WS children. These findings allow clinicians to identify the children most at risk of developing an anxiety disorder, and implications for early intervention programmes aimed at reducing anxiety risk in WS individuals. Further, based on the present findings, it is important to determine prior diagnoses of cardiovascular disease and levels of verbal IQ when seeking to identify at-risk children. Biological variables should also be considered with the finding that developmental trajectories differ according to sex.

## 6. Conclusions

The current study extends previous research by examining, for the first time, the prevalence and early patterns of anxiety in young children with WS. The key finding of the study was that anxiety symptomology is highly prevalent, even in very young children with WS, particularly GAD and specific phobia. Moreover, the prevalence of anxiety symptomology seems to increase with age over time, with many children developing comorbid anxiety disorder symptoms. Our longitudinal findings also provide evidence for the contribution of environmental factors on the nature, developmental course, and maintenance of anxiety. Chronological age, sex, and IQ were also found to impact the developmental trajectory of anxiety in young children with WS. Considerable individual variability was apparent, confirming the importance of individual assessments and developing individualised treatment programmes for those with WS. While the small sample size limits generalisability of findings, future research is needed to help determine which children are most at risk of developing an anxiety disorder and who should be targeted for early intervention. Although, with the very high prevalence rates indicated in the present study, and given the known link between genes implicated in WS and anxiety, perhaps all young children with WS would benefit from early preventative measures, if possible. For example, incorporating routine anxiety screening for all preschool WS children, and providing appropriate psychoeducation and training to parents and families of WS children seems warranted.

## Figures and Tables

**Table 1:** Demographic data for WS preschool children for Time 1 and Time 2 (*N* = 19).

	Time Point			
	Time 1		Time 2	
	Mean (*SD*)	Range	Mean (*SD*)	Range
CA (years) ^a^	4.08 (1.012)	2.24–5.97	7.53 (1.87)	3.98–9.90
Gender distribution	7 M, 12 F, 0 NB ^b^			
Family SES ^c^	3.59 (1.54)	1–5	3.59 (1.54)	1–5
Primary Caregiver Age (Years)	35.33 (3.46)	31–41	38.13 (3.87)	33–46
Global DQ ^d^	54.64 (11.71)	28.62–68.25	-	-
Verbal DQ ^d^	56.73 (16.50)	24.81–93.00	-	-
Nonverbal DQ ^d^	57.72 (13.83)	31.48–77.15	-	-
Current Interventions				
% Speech Therapy	100.00		100.00	
% Occupational Therapy	68.42		93.33	
% Physiotherapy	84.21		46.67	
% Psychological/Behaviour Therapy	0.00		15.79 ^d^	

Note. ^a^ chronological age. ^b^ NB = nonbinary. ^c^ SES = socioeconomic status as measured by the Australian Bureau of Statistics Index of Relative Socio-economic Advantage and Disadvantage [84] based on geographic residence. Families were provided with a score from “1” to “5”, with a lower score denoting a greater socioeconomic disadvantage. ^d^ DQ = developmental quotient as measured by MSEL (population *M* = 100, *SD* = 15; Scores 79 and below are considered impaired). ^d^ One child was receiving intervention for anxiety and aggression, a second child was receiving intervention for anxiety, and a third was receiving intervention for low mood.

**Table 2:** Anxiety symptoms at Time 1 and Time 2 (z scores).

Anxiety/Specific Phobia Subscales And Total Scores	Time Point								
	Time 1			Time 2 ^a^			Difference (T2-T1)		
	M (*SD*)	Range	% in Clinical Range ^e^	M (*SD*)	Range	% in Clinical Range ^e^	*T (18)*	*p*	*d*
Total Anxiety	−0.38 (0.59)	−1.17–0.44	0%	0.79 (1.31)	−1.21–**4.46**	31.58%	4.16	0.001 **	0.95
GAD ^b^	0.37 (1.18)	−0.94–**2.64**	36.84%	**1.22** (1.66)	−1.11–**4.68**	47.37%	2.52	0.021 *	0.58
Social Anxiety	−1.00 (0.35)	−1.19–0.12	0%	−0.39 (0.67)	−1.50–0.69	0%	21.00 ^d^	0.003 **	0.48 ^g^
OCD ^c^	−0.32 (0.72)	−0.67–**2.24**	5.26%	0.44 (1.16)	−0.71–**3.69**	31.58%	20.00 ^d^	0.003 **	0.49 ^g^
Physical Injury Fears	0.12 (0.89)	−1.42–**1.86**	15.79%	**1.19** (1.32)	−0.79–**3.96**	52.63%	3.58	0.002 **	0.82
Separation Anxiety	−0.11 (0.78)	−1.03–**1.52**	10.53%	0.90 (1.20)	−1.03–**4.24**	42.11%	3.76	0.001 **	0.86
Specific Phobia Total ^f^			63.16%			84.21%			
Animal			5.26%			26.32%			
Natural Environment			5.26%			21.05%			
Blood-Injectional-Injury			10.53%			26.32%			
Situational			26.53%			47.37%			
Other			15.79%			10.53%			
Loud Noises			36.84%			52.63%			

Note. *z* scores were utilised for all anxiety data analyses, with scores of one standard deviation above the mean for each subscale or Total Score of the community sample are considered clinically elevated (in line with [98]). ^a^ Combined PAS and SCAS-P scores. ^b^ GAD = Generalised Anxiety Disorder. ^c^ OCD = Obsessive–Compulsive Disorder. ^d^ Wilcoxon Signe-Rank Test utilised. ^e^ Percentage of total sample of young WS children in the clinical range (i.e., above the clinical cut-off of 1 *SD* above group mean of community sample). Bold typeface indicates mean *z* scores fell in the clinically elevated range. ^f^ Percentage of the total young children with WS who displayed at least one specific phobia. ^g^ effect size of r as Wilcoxon Signe-Rank Test utilised. * *p* < 0.05. ** *p* < 0.01.

**Table 3:** Anxiety Symptoms (including Specific Phobia) and Environmental factors Experienced by Individual WS children.

		Clinically Elevated Anxiety Symptoms ^a^		Significant Life Event ^b^		Additional Psychological Symptoms or Diagnosis ^c^		Psychological Intervention			
ID	Sex	Time 1	Time 2	Before Time 1	Between Time 1 and 2	Before Time 1	Between Time 1 and 2	Before Time 1	Between Time 1 and 2	Hyperacusis	Cardiovascular Abnormalities
1	M	None	GAD	Multiple hospital visits and surgeries	Started school	Problems concentrating; restless; fidgety	Problems concentrating; restless; fidgety	None	None	Yes	Yes
2	F	GAD; SP-blood-injury-injection; SP-Situational; SP-loud noises	GAD, OCD, Physical Injury Fears, Separation Anxiety; SP-Natural Environment; SP-blood-injury-injection; SP-Situational; SP-loud noises	Started school	New sibling	Problems concentrating; will not sleep alone	Problems concentrating; restless; fidgety	None	None	Yes	None
3	F	GAD; Separation Anxiety; SP-blood-injury-injection; SP-loud noises	OCD, SP-loud noises	Started preschool	Started school	Problems concentrating; restless; fidgety Repetitive thoughts; worries; trouble sleeping when separated from parents at night; hard to settle	Problems concentrating; restless; fidgety Repetitive thoughts; worries; trouble sleeping when separated from parents at night; hard to settle	None	None	Yes	Yes
4	F	None	SP-blood-injury-injection; SP-Situational; SP-Other; SP-loud noises	None	None	None	None	None	**Therapy for anxiety** and aggression	Yes	Yes
5	F	GAD; Physical; SP-loud noises	Physical Injury Fears; SP-Animals; SP-Natural Environment; SP-blood-injury-injection; SP-Other; SP-Loud Noises	Started preschool	Started school	Problems concentrating; hard to settle; depressed mood	Problems concentrating	None	None	Yes	Yes
6	F	GAD; SP-Situational	GAD; Physical Injury Fears; Separation Anxiety; SP-Animal; SP-Situational	Started school	None	Problems concentrating; restless; fidgety; hard to settle	**ADHDdiagnosis**; depressed mood	None	None	Yes	Yes
7	F	SP-Situational	Physical Injury Fears; SP-Situational	Started day-care	Started school; new Sibling	None	Problems concentrating; restless; fidgety	None	None	Yes	Yes
8	M	SP-Other	SP-Animals; SP-Natural Environment; SP-Situational	None	Started school; surgery	Problems concentrating; restless; fidgety; hard to settle	Problems concentrating; restless; fidgety; hard to settle	None	**Therapy for anxiety**	Yes	Yes
9	F	OCD	GAD	Started preschool	Started school	Restless; fidgety; hard to settle		None	None	Yes	Yes
10	F	None	Physical Injury Fears; Separation Anxiety	Started preschool	Started school	Restless; fidgety; hard to settle		None	None	Yes	Yes
11	M	GAD	GAD; OCD; Physical Injury Fears; Separation Anxiety; SP-Animals; SP-Loud Noises	Started preschool	None	Problems concentrating; restless; fidgety; hard to settle	Problems concentrating; restless; fidgety; hard to settle	None	None	Yes	Yes
12	M	GAD; Physical Injury Fears; SP-Natural Environment; SP-Other,	GAD; Separation Anxiety; SP-Other, SP-Situational; SP-Loud Noises	Started day-care	Parent Separation; started school	None	None	None	Therapy for mood	Yes	Yes
13	F	SP-Loud Noises	Separation Anxiety; SP- blood-injury-injection; SP-Loud Noises	Started school	None	Problems concentrating; restless; fidgety	Mild problems with concentration	None	None	Yes	None
14	F	SP-Situational; SP-Other	SP-Situational	None	Started Day-care	Problems concentrating	Problems concentrating; restless; fidgety	None	None	Yes	None
15	M	Physical Injury Fears; SP-Animal; SP-Loud Noises	Physical Injury Fears; Separation Anxiety; SP-Animal; SP-Natural Environment; SP-Situational; SP-Loud Noises	Started day-care	Started school	Hard to settle	None	None	None	Yes	Yes
16	F	GAD; Separation Anxiety; SP-Situational; SP-Loud Noises	GAD; OCD; Physical Injury Fears; SP-Situational; SP-Loud Noises	Separation of parents, started preschool	Started school	Problems concentrating; restless; fidgety; hyperactivity, hard to settle	Problems concentrating; restless; fidgety; hyperactivity,	None	None	Yes	Yes
17	M	SP-Loud Noises	GAD; OCD; Physical Injury Fears; Separation Anxiety; SP-Loud Noises	Surgery, started school	None	Problems concentrating; restless; fidgety	Problems concentrating; restless; fidgety	None	None	Yes	Yes
18	M	None	GAD; OCD; Physical Injury Fears; Separation; SP-Situational	Multiple surgeries, started school	None	Problems concentrating; restless; fidgety	Problems concentrating; restless; fidgety	None	None	Yes	Yes
19	F	None	Physical Injury Fears; Separation Anxiety; SP-Situational	Multiple surgeries, started preschool	Started school	Hard to settle	None	None	None	No	Yes

Note. ^a^ 1 SD above the ‘total’ group mean of the community sample as an indicator of clinical significance on the PAS and SCAS-P (as suggested by [98]; PAS was utilised for Time 1 and combined PAS and SCAS-P data for Time 2). ^b^ Significant life event experienced (both normative and non-normative). ^c^ Additional psychological symptoms and diagnoses that are not related to the listed anxiety symptoms. SP = Specific Phobia, GAD = Generalised Anxiety Disorder, OCD = Obsessive–Compulsive Disorder.

**Table 4:** Correlations Between the Change in Anxiety Over Time and Sex, Chorological Age, and DQ.

Anxiety Subtypes and Total Score	Chronological Age	Sex	DQ		
			Global	Verbal	Nonverbal
Total Score ^a^	0.13	0.02	−0.16	−0.34	−0.20
GAD ^b^	0.02	−0.41	−0.13	−0.34	−0.14
Social Anxiety	0.49 *	0.22	−0.06	−0.17	−0.20
OCD ^c^	−0.02	−0.24	0.30	0.11	0.21
Physical Injury Fears	0.31	0.61 **	−0.18	−0.14	−0.07
Separation Anxiety	0.36	−0.19	−0.11	−0.04	0.08

Note. ^a^ Scores represent Spearman’s Rho correlation coefficient. The ‘change in anxiety’ was calculated as the difference between the corresponding PAS and SCAS-P *z* scores at Time 1 and Time 2. ^b^ GAD = Generalised Anxiety Disorder. ^c^ OCD = Obsessive–Compulsive Disorder. Bold typeface indicates significant correlations. Bold typeface indicates moderate and large effect sizes. * *p* < 0.05. ** *p* < 0.01.

## Data Availability

Data is not available to share due to ethical reasons, as consent was not granted for this purpose.

## Appendix A

All 14 TD children were enrolled in an ongoing longitudinal study investigating neuropsychological functioning conducted at Macquarie University. They were recruited through the Macquarie University Neuronauts Brain Science Club and two of the child-care centres on campus at Macquarie University, Sydney, Australia. Children were screened for a history of psychological, developmental, and neurological conditions, and major sensory impairments that would impact on their ability to perform the research tasks. No child was excluded from this study based on these criteria. The chronological age range for this community comparison group was 2.21 to 2.98 years (*M* = 2.60, *SD* = 0.25).

At the time of testing, one TD child was receiving speech therapy intervention. Socioeconomic status (SES) of the families was quantified using the Australian Bureau of Statistics Index of Relative Socio-economic Advantage and Disadvantage (ABS, 2016) based on geographic residence. Families were provided with a score from “1” to “5”, with a lower score denoting a greater socioeconomic disadvantage. The mean score for the 2-year-old TD families was 4.38 (range 1 to 5), with a standard deviation of 1.33. Mothers were reported as being their child’s primary caregiver and, on average, completed 16.14 years of education (range 12 to 20 years; *SD* = 2.57). Demographic information regarding each TD family’s ethnic background revealed 64% identified as being Oceanic, with the remainder being Asian or European. Further demographic data and descriptive statistics for the TD sample are shown below in Table A1.

**Table A1:** Year-Old Typically Developing Community Comparison Group (*N* = 14).

	Mean (*SD*)	Range
CA (years) ^a^	2.60 (0.25)	2.21–2.98
Gender distribution	7 M, 7 F	
Overall DQ ^b^	109.45 (20.15)	72.17–151.47
Verbal DQ ^b^	107.15 (20.48)	63.81–141.88
Nonverbal DQ ^b^	106.31 (17.83)	80.52–136.01
Primary Caregiver Age (Years)	35.11 (5.78)	27–44
PAS (raw scores)		
Total Anxiety	12.64 (9.96)	1–33
GAD ^c^	1.14 (2.11)	0–8
Social Anxiety	3.64 (3.13)	0–10
OCD ^d^	1.21 (1.81)	0–6
Physical Injury Fears	3.50 (3.00)	0–11
Separation Anxiety	3.07 (3.20)	0–12

Note. ^a^ chronological age. ^b^ DQ = developmental quotient as measured by MSEL. ^c^ GAD = Generalised Anxiety Disorder. ^d^ OCD = Obsessive–Compulsive Disorder.

## References

[1] Kozel B.A., Barak B., Kim C., Mervis C.B., Osborne L.R., Porter M., Pober B.R. (2021). Williams syndrome. Nat. Rev. Dis..

[2] Dodd H.F., Porter M.A. (2009). Psychopathology in Williams syndrome: The effect of individual differences across the life span. J. Ment. Health Res. Intellect. Disabil..

[3] Leyfer O.T., Woodruff-Bordon J., Klein-Tasman B.P., Fricke J.S., Mervis C.B. (2006). Prevalence of psychiatric disorders in 4 to 6-year-olds with Williams syndrome. Am. J. Med. Genet. B Neuropsychiatr. Genet..

[4] Ng-Cordell E., Hanley M., Kelly A., Riby D.M. (2018). Anxiety in Williams syndrome: The role of social behaviour, executive functions, and change over time. J. Autism Dev. Disord..

[5] Davies M., Udwin O., Howlin P. (1998). Adults with Williams syndrome: Preliminary study if social, emotional and behavioural difficulties. Br. J. Psychiatry.

[6] Leyfer O., Woodruff-Borden J., Mervis C.B. (2009). Anxiety disorders in children with Williams syndrome, their mothers, and their siblings: Implications for the etiology of anxiety disorders. J. Neurodev. Disord..

[7] Plissart L., Borghgraef M., Volcke P., Van den Berghe H., Fryns J.P. (1994). Adults with Williams-Beuren syndrome: Evaluation of the medical, psychological and behavioural aspects. Clin. Genet..

[8] Riby D.M., Hanley M., Kirk H., Clark F., Little K., Fleck R., Janes E., Kelso L., O’Kane F., Cole-Fletcher R. (2014). The interplay between anxiety and social functioning in Williams syndrome. J. Autism Dev. Disord..

[9] Udwin O., Howlin P., Davies M., Mannion E. (1998). Community care for adults with Williams syndrome: How families cope and the availability of support networks. JIDR.

[10] Giora A., Gega L., Landau S., Marks I. (2005). Adult recall of having been bullied in attenders of an anxiety disorder unit and attenders of a dental clinic: A pilot controlled study. Behav. Change.

[11] La Greca A.M., Harrison H.M. (2005). Adolescent peer relations, friendships, and romantic relationships: Do they predict social anxiety and depression?. J. Clin. Child. Adolesc. Psychol..

[12] Strauss C.C., Frame C.L., Forehand R. (1987). Psychosocial impairment associated with anxiety in children. J. Clin. Neuropsychol..

[13] Cole D.A., Peeke L.G., Martin J.M., Truglio R., Seroczynski A.D. (1998). A longitudinal look at the relation between depression and anxiety in children and adolescents. J. Consult. Clin. Neuropsyhol.

[14] Stinton C., Elison S., Howlin P. (2010). Mental health problems in adults with Williams syndrome. Am. J. Intellect. Dev. Disabil..

[15] Caspi A., Elder G.H., Bem D.J. (1988). Moving away from the world: Life-course patterns of shy children. Dev. Psychol..

[16] Last C.G., Hansen C., Franco N. (1997). Anxious children in adulthood: A prospective study of adjustment. J. Am. Acad. Child Adolesc. Psychiatry.

[17] Fryssira H., Palmer R., Hallidie-Smith K.A., Taylor J., Donnai D., Reardon W. (1997). Flourescent in situ hybridisation (FISH) for hemizygous deletion at the elastin locus in patients with isolated supravalvar aortic stenosis. J. Med. Genet..

[18] Dykens E.M. (2000). Annotation: Psychopathology in children with intellectual disability. J. Child. Psychol. Psychiatry.

[19] Jabbi M., Kippenhan J.S., Kohn P., Marenco S., Mervis C.B., Morris C.A., Meyer-Lindenberg A., Berman K.F. (2012). The Williams syndrome chromosome 7q11. 23 hemideletion confers hypersocial, anxious personality coupled with altered insula structure and function. Proc. Natl. Acad. Sci. USA.

[20] Royston R., Howlin P., Waite J., Oliver C. (2017). Anxiety disorders in Williams syndrome contrasted with intellectual disability and the general population: A systematic review and meta-analysis. J. Autism Dev. Disord..

[21] Dykens E.M. (2003). Anxiety, Fears, and Phobias in persons with Williams syndrome. Dev. Neuropsychol..

[22] Meyer-Lindenberg A., Hariri A.R., Munoz K.E., Mervis C.B., Mattay V.S., Morris C.A., Berman K.F. (2005). Neural correlates of genetically abnormal social cognition in Williams syndrome. Nat. Neurosci..

[23] Meyer-Lindenberg A., Mervis C.B., Faith Berman K. (2006). Neural mechanisms in Williams syndrome: A unique window to genetic influences on cognition and behaviour. Nat. Rev. Neurosci..

[24] Muñoz K.E., Meyer-Lindenberg A., Hariri A.R., Mervis C.B., Mattay V.S., Morris C.A., Berman K.F. (2010). Abnormalities in neural processing of emotional stimuli in Williams syndrome vary according to social vs. non-social content. Neuroimage.

[25] Haas B.W., Mills D., Yam A., Hoeft F., Bellugi U., Reiss A. (2009). Genetic influences on sociability: Heightened amygdala reactivity and event-related responses to positive social stimuli in Williams syndrome. J. Neurosci..

[26] Dodd H.F., Porter M.A. (2010). I see happy people: Attention bias towards happy but not angry facial expressions in Williams syndrome. Cogn. Neuropsychiatry.

[27] Avery S.N., Thornton-Wells T.A., Anderson A.W., Urbano Blackfors J. (2012). White matter integrity deficits in prefrontal-amygdala pathways in Williams syndrome. Neuroimage.

[28] Thornton-Wells T.A., Avery S.N., Blackford J.U. (2011). Using novel control groups to dissect the amygdala’s role in Williams syndrome. Dev. Cogn. Neurosci..

[29] Rapee R.M., Schniering C.A., Hudson J.L. (2009). Anxiety disorders during childhood and adolescence: Origins and treatment. Annu. Rev. Clin. Psychol..

[30] Lieb R., Wittchen H.U., Höfler M., Fuetsch M., Stein M.B., Merikangas K.R. (2000). Parental psychopathology, parenting styles, and the risk of social phobia in offspring: A prospective-longitudinal community study. Arch. Gen. Psychiatry.

[31] Ehringer M.A., Rhee S.H., Young S., Corley R., Hewitt J.K. (2006). Genetic and environmental contributions to common psychopathologies of childhood and adolescence: A study of twins and their siblings. J. Abnorm. Child. Psychol..

[32] Gregory A.M., Eley T.C. (2007). Genetic influences on anxiety in children: What we’ve learned and where we’re heading. Clin. Child. Fam. Psychol. Rev..

[33] Thapar A., McGuffin P. (1995). Are anxiety symptoms in childhood heritable?. J. Child Psychol. Psychiatry Allied Discip..

[34] Smoller J.W. (2016). The genetics of stress-related disorders: PTSD, depression, and anxiety disorders. Neuropsychopharmacology.

[35] Sakurai K., Nishi A., Kondo K., Yanagida K., Kawakami N. (2011). Screening performance of K6/K10 and other screening instruments for mood and anxiety disorders in Japan. Psychiatry Clin. Neurosci..

[36] Schubert C. (2009). The genomic basis of the Williams–Beuren syndrome. Cell Mol. Sci..

[37] Hudson J.L., Rapee R.M., Antony M.M., Stein M.B. (2009). Familial and social environments in the etiology and maintenance of anxiety disorders. Oxford Handbook of Anxiety and Related Disorders.

[38] Green T., Avda S., Dotan I., Zarchi O., Basel-Vanagaite L., Zalsman G., Weizman A., Gothelf D. (2012). Phenotypic psychiatric characterization of children with Williams syndrome and response of those with ADHD to methylphenidate treatment. Am. J. Med. Genet. B Neuropsychiatr. Genet..

[39] Woodruff-Borden J., Kistler D.J., Henderson D.R., Crawford N.A., Mervis C.B. (2010). Longitudinal course of anxiety in children and adolescents with Williams syndrome. Am. J. Med. Genet. C Semin. Med. Genet..

[40] Uljarević M., Labuschagne I., Bobin R., Atkinson A., Hocking D.R. (2018). Brief report: The impact of sensory hypersensitivity and intolerance of uncertainty on anxiety in Williams syndrome. J. Autism Dev. Disord..

[41] Gagliardi C., Martelli S., Tavano A., Borgatti R. (2011). Behavioural features of Italian infants and young adults with Williams–Beuren syndrome. J. Intellect. Disabil. Res..

[42] Hahn L.J., Fidler D.J., Hepburn S.L. (2014). Adaptive behavior and problem behavior in young children with Williams syndrome. Am. J. Intellect. Dev. Disabil..

[43] Klein-Tasman B.P., Lee K. (2017). Problem behaviour and psychosocial functioning in young children with Williams syndrome: Parent and teacher perspectives. J. Intellect. Disabil. Res..

[44] Braga A.C., Carreiro L.R.R., Tafla T.L., Ranalli N.M.G., Honjo R.S., Kim C., Teixeira M.C.T.V. (2018). Cognitive and behavioral profile of Williams syndrome toddlers. CoDAS.

[45] Neo W.S., Tonnsen B.L. (2019). Brief Report: Challenging Behaviors in Toddlers and Preschoolers with Angelman, Prader–Willi, and Williams Syndromes. J. Autism Dev. Disord..

[46] Murthy R.S. (2007). Mass violence and mental health: Recent epidemiological findings. Int. Rev. Psychiatry.

[47] Yule W., Udwin O., Murdoch K. (1990). The ‘Jupiter’ sinking: Effects on children’s fears, depression and anxiety. J. Child Psychol. Psychiatry Allied Discip..

[48] Eley T.C., Stevenson J. (2000). Specific life events and chronic experiences differentially associated with depression and anxiety in young twins. J. Abnorm. Child. PSychol.

[49] Eley T.C., Lau J.Y., Hudson J., Rapee R. (2005). Genetics and the family environment. Psychopathology and the Family.

[50] Broeren S., Muris P., Diamantopoulou S., Baker J.R. (2013). The course of childhood anxiety symptoms: Developmental trajectories and child-related factors in normal children. J. Abnorm. Child. Psychol..

[51] Mian N.D., Wainwright L., Briggs-Gowan M.J., Carter A.S. (2011). An ecological risk model for early childhood anxiety: The importance of early child symptoms and temperament. J. Abnorm. Child. Psychol..

[52] Thomas A., Chess S. (1977). Temperament and Development.

[53] Hudson J.L., Rapee R.M., Heimberg R.G., Turk C.L., Mennin D.S. (2004). From Anxious Temperament to Disorder: An Etiological Model. Generalized Anxiety Disorder: Advances in Research and Practice.

[54] Khreim I., Mikkelsen E. (1997). Anxiety disorders in adults with mental retardation. Psychiatr. Ann..

[55] American Psychiatric Association (2013). Diagnostic and Statistical Manual of Mental Disorders.

[56] World Health Organisation (1992). ICD-10 Classifications of Mental and Behavioural Disorders: Clinical Descriptions and Diagnostic Guidelines.

[57] March J.S., Parker J.D., Sullivan K., Stallings P., Conners C.K. (1997). The Multidimensional Anxiety Scale for Children (MASC): Factor structure, reliability, and validity. J. Am. Acad. Child Adolesc. Psychiatry.

[58] Muris P., de Jong P.J., Engelen S. (2004). Relationships between neuroticism, attentional control, and anxiety disorders symptoms in non-clinical children. Pers. Individ. Dif..

[59] Parker G., Hadzi-Pavlovic D. (2004). Is the female preponderance in major depression secondary to a gender difference in specific anxiety disorders?. Psychol. Med..

[60] Roza S.J., Hofstra M.B., Van Der Ende J., Verhulst F.C. (2003). Stable prediction of mood and anxiety disorders based on behavioral and emotional problems in childhood: A 14-year follow-up during childhood, adolescence, and young adulthood. Am. J. Psychiatry.

[61] King N.J., Ollier K., Iacuone R., Schuster S., Bays K., Gullone E., Ollendick T.H. (1989). Fears of children and adolescents: A cross-sectional Australian study using the revised-fear survey schedule for children. J. Child Psychol. Psychiatry Allied Discip..

[62] Einfeld S.L., Tonge B.J. (1996). Population prevalence of psychopathology in children and adolescents with intellectual disability: II epidemiological findings. J. Intellect. Disabil. Res..

[63] Ng R., Järvinen A., Bellugi U. (2014). Characterizing associations and dissociations between anxiety, social, and cognitive phenotypes of Williams syndrome. Res. Dev. Disabil..

[64] Porter M.A., Dodd H., Cairns D. (2009). Psychopathological and behavior impairments in Williams-Beuren syndrome: The influence of gender, chronological age, and cognition. Child. Neuropsych.

[65] Woodcock R.W., Johnson M.B. (1990). Woodcock-Johnson Psycho-Educational Battery—Revised.

[66] Osório A.A., Rossi N.F., Gonçalves Ó.F., Sampaio A., Giacheti C.M. (2017). Psychopathology and behavior problems in children and adolescents with Williams syndrome: Distinctive relationships with cognition. Child. Neuropsych.

[67] Achenbach T.M., Rescorla L.A. (2001). Manual for the ASEBA School-Age Forms & Profiles.

[68] Rzepecka H., McKenzie K., McClure I., Murphy S. (2011). Sleep, anxiety and challenging behaviour in children with intellectual disability and/or Autism Spectrum Disorder. Res. Dev. Disabil..

[69] Silverman W.K., Albano A.M. (1996). Anxiety Disorders Interview Schedule for DSM-IV: Child Version.

[70] Kaufman J., Birmaher B., Brent D., Rao U., Flynn C., Moreci P., Williamson D., Ryan N. (1997). Schedule for affective disorders and schizophrenia for school-aged children-present and lifetime version (K-SADS-PL): Initial reliability and validity data. J. Am. Acad. Child Adolesc. Psychiatry.

[71] American Psychiatric Association (1994). Diagnostic and Statistical Manual of Mental Disorders: DSM-IV.

[72] Spence S.H. (1997). Structure of anxiety symptoms among children: A confirmatory factor-analytic study. J. Abnorm. Psychol..

[73] Papaeliou C., Polemikos N., Fryssira E., Kodakos A., Kaila M., Yiota X., Benaveli C., Michaelides V., Stroggilos M., Vrettopoulou M. (2012). Behavioural profile and maternal stress in Greek young children with Williams syndrome. Child Care Health Dev..

[74] Achenbach T.M., Rescorla L.A. (2000). Manual for the ASEBA Preschool Forms & Profiles.

[75] Ferdinand R.F. (2008). Validity of the CBCL/YSR DSM-IV scales anxiety problems and affective problems. J. Anxiety Disord..

[76] Korenberg J.R., Chen X.N., Hirota H., Lai Z., Bellugi U., Burian D., Roe B., Matsuoka R. (2000). VI. Genome structure and cognitive map of Williams syndrome. J. Cogn. Neurosci..

[77] Tassabehji M., Metcalfe K., Fergusson W.D., Carette M.J., Dore J.K., Donnai D., Reed A.P., Pröschel C., Gutowski N.J., Mao X. (1996). LIM–kinase deleted in Williams syndrome. Nat. Genet..

[78] Mullen E.M. (1995). Mullen Scales of Early Learning.

[79] Mervis C.B., Robinson B.F., Bertrand J., Morris C.A., Klein-Tasman B.P., Armstrong S.C. (2000). The Williams syndrome cognitive profile. Brain Cogn..

[80] Miezah D., Porter M., Batchelor J., Boulton K., Veloso G.C. (2020). Cognitive abilities in Williams syndrome. Res. Dev. Disabil..

[81] Miezah D., Porter M., Rossi A., Kazzi C., Batchelor J., Reeve J. (2021). Cognitive profile of young children with Williams syndrome. J. Intellect. Disabil. Res..

[82] Martens M.A., Wilson S.J., Reutens D.C. (2008). Research review: Williams syndrome: A critical review of the cognitive, behavioral, and neuroanatomical phenotype. J. Child. Psychol. Psychiatry.

[83] Porter M.A., Coltheart M. (2005). Cognitive heterogeneity in Williams syndrome. Dev. Neuropsychol..

[84] Australian Bureau of Statistics. https://www.abs.gov.au/ausstats/abs@.nsf/Lookup/by%20Subject/2033.0.55.001~2016~Main%20Features~IRSAD%20Interactive%20Map~16.

[85] Spence S.H., Rapee R., McDonald C., Ingram M. (2001). The structure of anxiety symptoms among preschoolers. Behav. Res. Ther..

[86] Galán-Luque T., Serrano-Ortiz M., Orgilés M. (2025). Factor Structure and Psychometric Properties of the Spence Children’s Anxiety Scale: A 25-Year Systematic Review. Child. Psychiatry Hum. Dev..

[87] Nauta M.H., Scholing A., Rapee R.M., Abbot M., Spence S.H., Waters A. (2004). A parent-report measure of children’s anxiety: Psychometric properties and comparison with child-report in a clinic and normal sample. Behav. Res. Ther..

[88] Rodgers J., Riby D.M., Janes E., Connolly B., McConachie H. (2012). Anxiety and repetitive behaviours in Autism Spectrum Disorders and Williams syndrome: A cross-syndrome comparison. J. Autism Dev. Disord..

[89] Spence S.H., Rapee R. (1999). Preschool Anxiety Scale (Parent Report).

[90] Kazzi C., Porter M., Zhong Q., Veloso G., Reeve J. (2021). The relationship between anxiety and executive functioning in children with Williams syndrome. Glob. J. Intellect. Dev. Disabil..

[91] Spence S.H. (1998). A measure of anxiety symptoms among children. Behav. Res. Ther..

[92] Rogers S.J., Estes A., Lord C., Vismara L., Winter J., Fitzpartick A., Guo M., Dawson G. (2012). Effects of brief Early Start Denver Model (ESDM)-based parent intervention on toddlers at risk for autism spectrum disorders: A random controlled trial. J. Am. Acad. Child Adolesc. Psychiatry.

[93] Campbell L.E., Daley E., Toal F., Stevens A., Azuma R., Karmiloff-Smith A., Murphy D.G.M., Murphy M.K.C. (2009). Brain structural differences associated with the behavioural phenotype in children with Williams syndrome. Brain Res..

[94] Hocking D.R., Reeve J., Porter M.A. (2015). Characterising the profile of everyday executive functioning and relation to IQ in adults with Williams syndrome: Is the BRIEF adult version a valid rating scale?. PLoS ONE.

[95] Rhodes S.M., Riby D.M., Park J., Fraser E., Campbell L.E. (2010). Executive neuropsychological functioning in individuals with Williams syndrome. Neuropsychologia.

[96] Rothman K.J. (1990). No adjustments are needed for multiple comparisons. Epidemiology.

[97] Cohen J. (1988). Statistical Power Analysis for the Behavioural Sciences.

[98] The Spence Children’s Anxiety Scale. SCAS. https://www.scaswebsite.com/.

[99] McAdams P.D. (1985). Intimacy, and the Life Story: Personological Inquiries into Identity.

[100] Pillemer D.B. (2001). Momentous events and the life story. Rev. Gen. Psychol..

[101] Baxter A.J., Scott K.M., Vos T., Whiteford H.A. (2013). Global prevalence of anxiety disorders: A systematic review and meta-regression. Psychol. Med..

[102] Somers J.M., Goldner E.M., Waraich P., Hsu L. (2006). Prevalence and incidence studies of anxiety disorders: A systematic review of the literature. Can. J. Psychiatry.

[103] Dennis M., Landry S.H., Barnes M., Fletcher J.M. (2006). A model of neurocognitive function in spina bifida over the life span. JINS.

[104] Luby J.L. (2013). Treatment of anxiety and depression in the preschool period. J. Am. Acad. Child. Adolesc..

[105] Connolly S.D., Bernstein G.A. (2007). Practice parameter for the assessment and treatment of children and adolescents with anxiety disorders. J. Am. Acad. Child. Adolesc..

[106] Finelli J., Gleason M.M., Frankel K., Harrison J., Njoroge W. (2019). Psychopharmacologic considerations in early childhood. Clinical Guide to Psychiatric Assessment of Infants and Young Children.

[107] Zaim N., Harrison J. (2020). Pre-school mental health disorders: A review. Int. Rev. Psychiatry.

[108] Fynn G., Porter M., Borchard T., Kazzi C., Zhong Q., Campbell L. (2023). The effectiveness of cognitive behavioural therapy for individuals with an intellectual disability and anxiety: A systematic review. JIDR.

[109] Wittchen H.U., Hoyer J. (2001). Generalized anxiety disorder: Nature and course. J. Clin. Psychiatry.

[110] Danesh A.A., Howery S., Aazh H., Kaf W., Eshraghi A.A. (2021). Hyperacusis in Autism Spectrum Disorders. Audiol. Res..

[111] Barrett P.M., Dadds M.R., Rapee R.M. (1996). Family treatment of childhood anxiety: A controlled trial. J. Consult. Clin. Psychol..

[112] Toscano R., Baillie A.J., Lyneham H.J., Kelly A., Kidd T., Hudson J.L. (2020). Assessment of anxiety in children and adolescents: A comparative study on the validity and reliability of the Spence Children’s Anxiety Scale in children and adolescents with anxiety and Autism Spectrum Disorder. J. Affect. Disord..

[113] Finlay W.M., Lyons E. (2002). Acquiescence in interviews with people who have mental retardation. Ment. Retard..

[114] Hartley S.L., MacLean W.E. (2006). A review of the reliability and validity of Likert-type scales for people with intellectual disability. JIDR.

[115] Hettema J.M., Neale M.C., Kendler K.S. (2001). A review and meta-analysis of the genetic epidemiology of anxiety disorders. Am. J. Psychiatry.

[116] Rapee R.M., Spence S.H. (2004). The etiology of social phobia: Empirical evidence and an initial model. Clin. Psych. Rev..

[117] Dai L., Carter C.S., Ying J., Bellugi U., Pournajafi-Nazarloo H., Korenberg J.R. (2012). Oxytocin and vasopressin are dysregulated in Williams Syndrome, a genetic disorder affecting social behavior. PLoS ONE.

[118] Griffiths H., Fazel M.S. (2016). Early intervention crucial in anxiety disorders in children. Practitioner.

[119] Huston J.C., Thom R.P., Ravichandran C.T., Mullett J.E., Moran C., Waxler J.L., Prober B.R., McDougle C.J. (2021). Repetitive Thoughts and Repetitive Behaviors in Williams Syndrome. J. Autism Dev. Disord..

